# Nasal symbiont *Staphylococcus epidermidis* restricts the cellular entry of influenza virus into the nasal epithelium

**DOI:** 10.1038/s41522-022-00290-3

**Published:** 2022-04-13

**Authors:** Ara Jo, Jina Won, Chan Hee Gil, Su Keun Kim, Kang-Mu Lee, Sang Sun Yoon, Hyun Jik Kim

**Affiliations:** 1 Department of Otorhinolaryngology, Seoul National University College of Medicine, Seoul, Republic of Korea; 2 Department of Microbiology and Immunology, Institute for Immunology and Immunological Diseases, Yonsei University College of Medicine, Seoul, Republic of Korea; 3 Seoul National University Hospital, Seoul, Republic of Korea; 4 Sensory Organ Research Institute, Seoul National University Medical Research Center, Seoul, Republic of Korea

**Keywords:** Microbiome, Symbiosis

## Abstract

Our recent study presented that human nasal commensal *Staphylococcus epidermidis* could potentiate antiviral immunity in the nasal mucosa through interferon-related innate responses. Here, we found that human nasal commensal *S. epidermidis* promoted protease–protease inhibitor balance in favor of the host and prevented influenza A virus (IAV) replication in the nasal mucosa and lungs. A relatively higher induction of Serpine1 exhibited in *S. epidermidis*-inoculated nasal epithelium and *S. epidermidis*-induced Serpine1 significantly decreased the expression of serine proteases. Furthermore, the transcription of urokinase plasminogen activator (uPA) and Serpine1 was biologically relevant in *S. epidermidis*-inoculated nasal epithelium, and the induction of uPA might be related to the sequential increase of Serpine1 in human nasal epithelium. Our findings reveal that human nasal commensal *S. epidermidis* manipulates the cellular environment lacking serine proteases in the nasal epithelium through Serpine1 induction and disturbs IAV spread to the lungs at the level of the nasal mucosa.

## Introduction

It is becoming increasingly apparent that the primary targets of respiratory viruses, including influenza viruses, are the respiratory epithelium and the innate immune system of the respiratory epithelium serves as the first-line defense mechanism to suppress respiratory virus infection^1–3^. The respiratory epithelium produces antiviral and chemotactic molecules that initiate immune responses by rapid recruitment of innate effector cells and have their own unique mechanisms to prime antiviral immune responses in the respiratory tract against influenza viruses^3^. Additionally, adequate immune interactions in respiratory epithelial cells are critical to disturb the cellular entry of influenza viruses and can prevent the spread of these viruses to the respiratory tract^4^. To enter into the airway epithelial cells for the initiation of virus infection, influenza viruses require the maturation cleavage of viral surface glycoproteins, and the maturation cleavage depends on host cell proteases for activation of the viral protein^5^. In this regard, the reduction of airway protease represents an attractive host-cell targeting antiviral strategy in the respiratory tract, and serine protease inhibitors have been reported to suppress influenza virus replication with the inhibition of intracellular invasion by targeting host serine proteases^6–8^.

The respiratory mucosa is in direct contact with the respiratory epithelium and constantly exposed to inhaled pathogens, which directly impact the mucosal immune mechanisms of the respiratory epithelium^9,10^. Importantly, the colonizing microbiome in the human respiratory mucosa is subject to mediate mucosal immune defense mechanisms, and studies on the reaction of the mucosal microbiome to the inhaled pathogens within the host increasingly consider the contribution of immune responses^11–14^. Inhaled respiratory viruses encounter the host’s immune system for the first time in the nasal passage, and the microbial characteristics of the nasal mucus are closely related to the mechanisms of initial immune responses^15,16^. Thus, insights into the microbiota of the human nasal mucosa can provide fundamental information regarding an individual’s susceptibility to respiratory viral infections and factors contributing to related immune mechanisms^17,18^. Our previous study identified that *Staphylococcus epidermidis* was the most abundant commensal present in the nasal mucus of healthy humans and showed that the *S. epidermidis* that were isolated from healthy nasal mucus accelerated the clearance of influenza virus from the nasal epithelium^15^. In addition, the inoculation of the human nasal commensal *S. epidermidis* into an in vivo nose limited influenza virus-caused lung infection via interferon-related innate immune responses. Complex host–microbe interactions have made it difficult to gain a detailed understanding of the mechanisms involved in the inhibition of colonization. However, it has been proven that the serine proteases secreted by a subset of *S. epidermidis* inhibits biofilm formation and nasal colonization of inhaled nasal pathogens^19^. In this study, we aimed to elucidate other antiviral mechanisms of *S. epidermidis* and prelude that *S. epidermidis*, a major symbiont in healthy nasal mucus, might have the ability to induce specific serine proteases, resulting in the activation of serine protease inhibitors. Here, our data uncover another capacity of *S. epidermidis* to potentiate antiviral innate immune responses in the nasal epithelium through the induction of the serine protease inhibitor Serpine1, driven by urokinase plasminogen activator. *S. epidermidis* creates an intracellular environment lacking serine proteases in the nasal epithelium and inhibits viral invasion into nasal epithelial cells. Our results indicate that a beneficial commensal *S. epidermidis* promotes protease–protease inhibitor balance in favor of the host nasal mucosa and provides a biological antiviral arsenal against influenza virus spread.

## Results

### Nasal microbiota may be critical for the suppression of influenza A virus (IAV)-caused lung infection

**Fig. 1:**
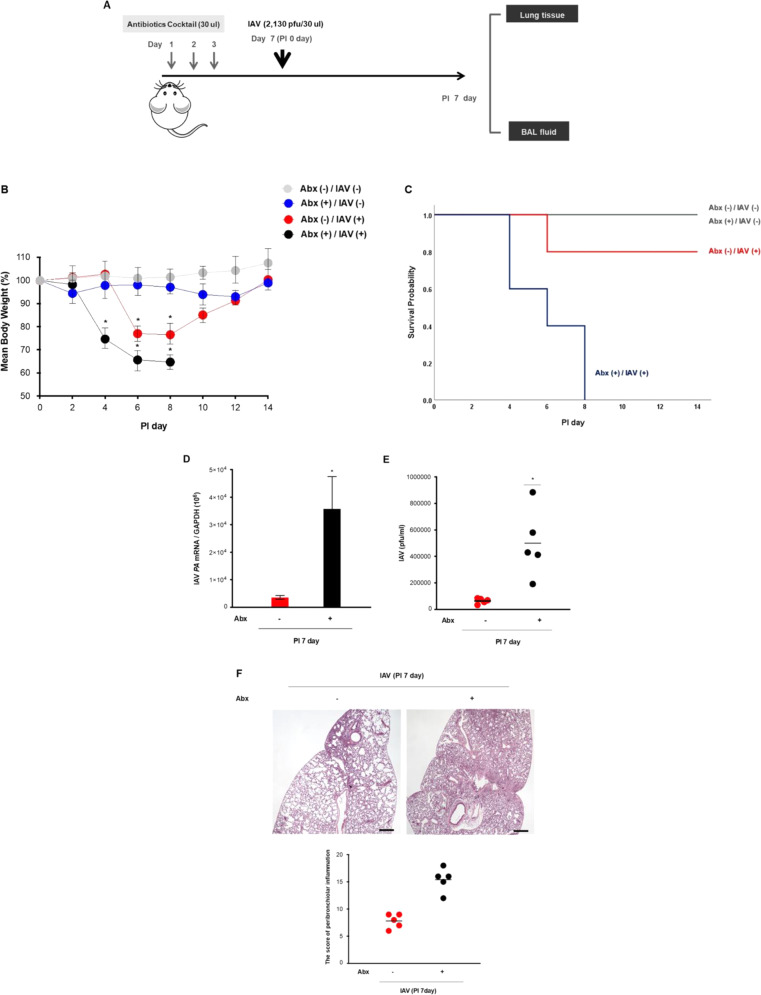
Nasal microbiota might be critical for the suppression of IAV-caused lung infection. **A** Schematic of the mouse model experimental design with depletion of the nasal microbiome. The mice (*N* = 20, group 1 (*n* = 5): abx (−) IAV (−), group2 (*n* = 5): abx (+) IAV (−), group 3 (*n* = 5): abx (−) IAV(+), group 4 (*n* = 5): abx (+) IAV (+)) were used in these experiments and ten mice were infected with IAV (2,130 pfu) at the indicated time points. The change in **B** the mean body weight (analyzed by repeated measure two-way ANOVA), and **C** survival rate of IAV-infected mice (analyzed by Kaplan–Meier with log-rank test) was compared according to the inoculation of intranasal antibiotics. Ten mice were used and five (abx (+), IAV (+)) were infected with IAV (2,130 pfu). The B6 mice with depletion of the nasal microbiota before IAV infection exhibited over 10% of weight loss than IAV-infected mice without abx administration, resulting in the death of all the mice at 8 days after IAV infection. Lung samples from uninfected mice (abx (+), IAV (−)) and IAV-infected mice that survived up to 7 dpi were collected. **D** IAV *PA* mRNA levels in the mice lung tissue (red dot: abx (+), IAV (–), black dot: abx (+) IAV (+)) and **E** viral titers from the BAL fluid of IAV-infected mice (red dot: abx (+), IAV (−), black dot: abx (+) IAV (+)) were assessed at 7 dpi. **F** H&E-stained micrographs were also generated from lung sections obtained at 7 dpi. Micrographs shown are representative of lung sections from five mice (red dot: abx (+), IAV (−), black dot: abx (+) IAV (+)) (Scale bar 10 uM). The micrographs were used to assess inflammation and tissue damage and to calculate a histological score. Real-time PCR and plaque assay results are analyzed by Mann–Whitney *U*-test and presented as mean ± SD values from three independent experiments. *p < 0.05 vs. mice without antibiotics treatment. (Abx antibiotics).

We first assessed whether the nasal microbiota could be important when considering its antiviral protective properties using a murine model of IAV-caused lung infection. The nasal cavities of C57BL/6 (B6) mice (*N* = 5) were inoculated with 30 μL of an antibiotic cocktail (composed of vancomycin, neomycin, ampicillin, and metronidazole) on days 1, 2, and 3 to eliminate nasal microbiota completely (Supplementary Fig. 1), followed by IAV [2,130 pfu/30 μL, phosphate-buffered saline (PBS)] infection on day 7 (Fig. 1A).

The body weights and survival rates of the infected B6 mice were monitored for 14 days. The IAV-infected mice exhibited a significant decrease in mean body weight, with an 80% survival rate until seven days postinfection (dpi) compared to the mice without antibiotic administration and IAV infection. The B6 mice with depletion of the nasal microbiota before IAV infection exhibited a more significant weight loss (below 15 g until 7 dpi) than IAV-infected mice without antibiotic administration, resulting in the death of all the mice after IAV infection (Fig. 1B, C). As compared with thee infected with IAV alone (3.2 × 10^3^), those inoculated with antibiotics preceding IAV infection showed higher IAV *polymerase acidic protein (PA)* gene levels (3.4 × 10^4^) in lung tissue (Fig. 1D) and higher viral titer (4.8 × 10^4^ with antibiotics) in bronchoalveolar lavage (BAL) fluid (Fig. 1E). IAV-infected mice with depletion of the nasal microbiota also had more severe pathologic findings in the lungs, with significantly higher histologic scores (Fig. 1F).

These findings demonstrate that IAV-caused lung infection progressed more seriously when the nasal microbiota was eliminated before infection and that the nasal microbiome could contribute to immune responses against IAV infection in the respiratory tract.

### Pretreatment with human nasal commensal *S. epidermidis* suppresses IAV-caused infection in the lungs and nasal mucosa in vivo

**Fig. 2:**
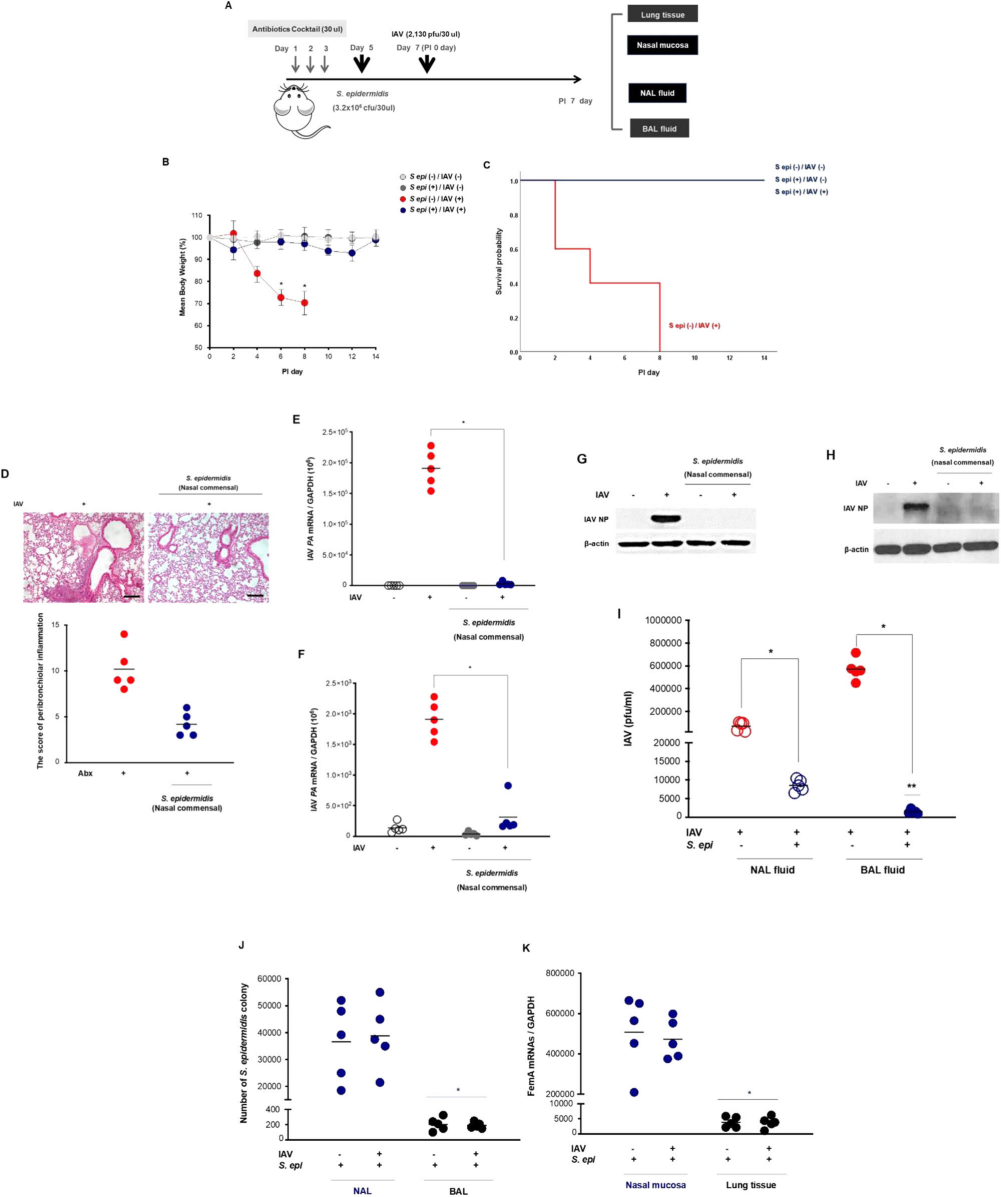
Human nasal mucus–derived *S. epidermidis* suppressed the viral spread and replication in IAV-infected mice. **A** Schematic of the mouse model experimental design for intranasal *S. epidermidis* inoculation and IAV infection. The mice (*N* = 20, group 1 (*n* = 5): *S. epidermidis* (−) IAV (−), group2 (*n* = 5): *S. epidermidis* (+) IAV (−), group 3 (*n* = 5): *S. epidermidis* (−) IAV (+), group 4 (*n* = 5): *S. epidermidis* (+) IAV (+)) were used in these experiments. The mice were inoculated with *S. epidermidis* (3.2 × 10^6^ CFU/30 µl PBS) and infected with IAV (2,130 pfu/30 ul PBS) at the indicated time points. The changes in (**B**) the mean body weight (analyzed by repeated measure two-way ANOVA) and survival rate (**C**) of IAV-infected mice (analyzed by Kaplan–Meier with log-rank test) were compared according to the inoculation of *S. epidermidis*. 20 mice were used and ten were infected with IAV (2,130 pfu). IAV-infected mice with depletion of the nasal microbiota exhibited over 30% decrease in mean body weight until eight days but the mean body weight of nasal microbiota-depleted B6 mice that were treated with *S. epidermidis* before IAV infection exceeded 20 g until 8 days after infection. Lung samples from uninfected mice and mice that survived up to 7 dpi were collected. **D** B6 mice were inoculated with human nasal mucus–derived *S. epidermidis* before IAV infection, and H&E-stained micrographs were also generated from lung sections obtained at 7 dpi (Scale bar 100 uM). Micrographs shown are representative of lung sections from five mice and were used to assess inflammation and tissue damage and to calculate a histological score. B6 mice were inoculated with human nasal *S. epidermidis* before IAV infection, and IAV *PA* mRNA levels in the mouse lung tissue 9 (**E**) and nasal mucosa (**F**) were assessed at 7 dpi. Levels of IAV NP were monitored in lung tissue (**G**) and nasal mucosa (**H**) using western blot analysis, and representative results are shown. **I** Viral titers were also measured in the BAL fluid of IAV-infected mice following *S. epidermidis* inoculation. In the next independent experiments, ten mice were used to measure the colonies and *FemA* mRNA levels of *S. epidermidis* according to IAV infection. **J** *S. epidermidis* CFUs were determined at 7 dpi in the NAL and BAL fluid of IAV-infected mice following *S. epidermidis* inoculation. **K** *FemA* mRNA levels of *S. epidermidis* were measured in the nasal mucosa and lung tissue of IAV-infected mice at 7 dpi following *S. epidermidis* inoculation. Real-time PCR, plaque assays, colony count, and ELISA results are analyzed by Mann–Whitney *U*-test and presented as mean ± SD values from three independent experiments. *p < 0.05 vs. mice infected with IAV alone.

We next sought to explore whether the human nasal commensal *S. epidermidis* exhibited antiviral protective properties against IAV infection in a murine model in vivo. The nasal cavities of B6 mice (*N* = 5) were inoculated with human nasal mucus–derived *S. epidermidis* on day 5, which was 2 days after depletion of the nasal microbiota (days 1, 2, and 3) using 30 μL of antibiotics. Then, the *S. epidermidis*–inoculated B6 mice were infected with IAV (2130 pfu/30 μl, PBS) 2 days after *S. epidermidis* inoculation (day 7) (Fig. 2A).

The results revealed that IAV-infected mice with depletion of the nasal microbiota exhibited a significant decrease in mean body weight with a 20% survival rate until 14 days. Interestingly, the nasal microbiota-depleted B6 mice that were treated with human nasal *S. epidermidis* before IAV infection maintained their body weight even after IAV infection, and the mean body weight of these mice exceeded 20 g until seven days after infection, resulting in a 100% survival rate after IAV infection as the mice without IAV infection and *S. epidermidis* inoculation (Fig. 2B, C). Nasal microbiota-depleted B6 mice with only an *S. epidermidis* intranasal inoculation did not show any weight loss and also exhibited a 100% survival rate. As compared with the IAV-infected mice with intranasal antibiotic administration, *S. epidermidis* exposure also resulted in attenuated pathologic findings in the lungs of IAV-infected mice, with significantly lower histologic scores (Fig. 2D).

Human nasal *S. epidermidis* also demonstrated antiviral potential by suppressing viral replication in the lungs and nasal mucosa of IAV-infected mice. The data revealed that B6 mice infected with IAV and inoculated with *S. epidermidis* had lower IAV *PA* messenger RNA (mRNA) levels (2.7 × 10^2^) in the lung tissue (Fig. 2E), and lower IAV *PA* mRNA levels (0.4 × 10^3^) were also observed in the nasal mucosa (Fig. 2F). Moreover, the IAV nucleoprotein (NP) level was significantly reduced in the lungs and nasal mucosa of IAV-infected mice that were inoculated with human nasal *S. epidermidis* relative to in the IAV-infected mice with depletion of the nasal microbiota (Fig. 2G, H). In addition, viral titers were also significantly reduced in the nasal lavage (NAL) and BAL fluid of IAV-infected mice with inoculation with human nasal *S. epidermidis* (Fig. 2I). While the viral titer was relatively higher in BAL fluid accompanied by higher IAV mRNA levels in the lung tissue of IAV-infected mice with depletion of the nasal microbiota, it was rather decreased a lesser extent in the NAL fluid of IAV-infected mice following inoculation of *S. epidermidis* despite the fact that IAV replication was suppressed in the nasal mucosa and the lungs.

As a next step, we assessed the distribution of bacteria in *S. epidermidis*-inoculated mice by comparing colony-forming units (CFUs) in NAL and BAL fluid. Whereas substantial numbers of *S. epidermidis* CFUs were observed in the NAL fluid, the levels of *S. epidermidis* cells in the BAL fluid were under the limits of detection (Fig. 2J). We also found that mRNA levels of the *FemA* gene in *S*. *epidermidis* were minimally detected in the lungs of mice after nasal inoculation with *S. epidermidis* (Fig. 2K).

These findings suggest that human nasal microbiome *S. epidermidis* suppresses IAV replication not only in nasal mucosa but only the lungs of IAV-infected mice and the number of extracellular IAV particles is estimated to be reduced to a lesser extent in NAL fluid, which is thought to be related to inhibition of cellular entry of the virus at the level of the nasal mucosa.

### Human nasal commensal *S. epidermidis* promotes the induction of serine protease inhibitors in the nasal epithelium

**Fig. 3:**
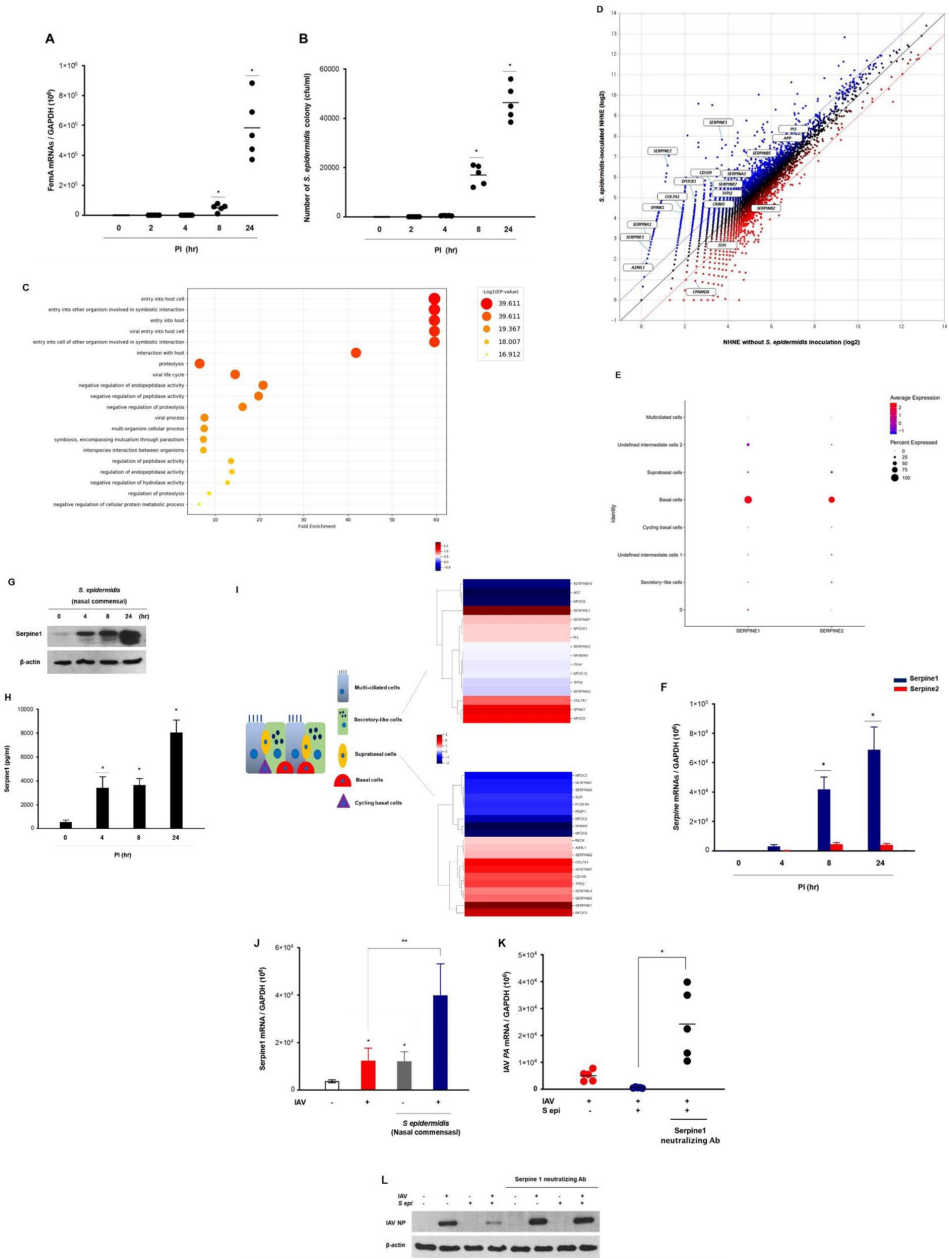
Human nasal commensal *S. epidermidis* promoted the induction of serine protease inhibitor in the nasal epithelium. NHNE cells from five healthy volunteers were inoculated with human nasal *S. epidermidis* at an MOI of 0.25. **A** *FemA* mRNA levels, normalized to cellular *GAPDH* transcript levels, were monitored by real-time PCR. **B** *S. epidermidis* CFUs were determined at 1 dpi in the supernatant of *S. epidermidis*-inoculated NHNE cells. **C** Dot plot visualization of enriched GO terms in *S. epidermidis–*inoculated NHNE cells at 1 dpi. **D** Scatterplots indicating enriched genes correlated with serine-type peptidase inhibitors. **E** Expressions of *Serpine1* and *Serpine2* from scRNA-seq of NHNE cells. Dot size represents the proportion of *Serpines* RNA expression within the respective cell type expressing the gene, and dot color represent the average *Serpines* RNA expression level in the particular cell type. **F** Serpine1 and Serpine2 mRNA levels, normalized to cellular *GAPDH* transcript levels, were monitored by real-time PCR (blue bar: Serpine1; red bar: Serpine2). **G** The intracellular protein level of Serpine1 was measured in the cell lysate of *S. epidermidis–*inoculated NHNE cells using western blot analysis. **H** The secreted protein level of Serpine1 was measured in the supernatant of *S. epidermidis*-inoculated NHNE cells using ELISA. I Heatmap depicting the expression levels of genes related with serine-type peptidase inhibitor activity differentially expressed in *S. epidermidis*-inoculated NHNE cells depending on the cellular subset. **J** Serpine1 mRNA level, normalized to cellular *GAPDH* transcript levels, was monitored by real-time PCR over 1 day after IAV infection following *S. epidermidis* inoculation. **K**, **L** The neutralizing antibody for Serpine1 was administered to NHNE cells one hour before *S. epidermidis* inoculation, and then the cells were infected with IAV for 1 day. IAV mRNA and NPs of IAV were compared in IAV-infected NHNE cells after *S. epidermidis* inoculation depending upon the neutralization of Serpine1. Results are presented as mean ± SD values from five independent experiments. *p < 0.05 vs. mock-infected NHNE cells.

To determine whether cellular entry of IAV might be suppressed in the nasal epithelium via human nasal commensal *S. epidermidis*, *S. epidermidis* were inoculated to normal human nasal epithelial (NHNE) cells. First, mRNA levels in the cell lysate and the colony count in the supernatant were assessed at different time points postinfection following the inoculation of *S. epidermidis* at a multiplicity of infection (MOI) of 0.25. Real-time polymerase chain reaction (PCR) revealed that *S. epidermidis FemA* mRNA levels increased significantly from eight hours postinfection, with the highest levels observed at 1 dpi (5.8 × 10^5^; Fig. 3A). The number of *S. epidermidis* CFUs was also significantly increased in the supernatant of *S. epidermidis*-inoculated NHNE cells until one day after inoculation (Fig. 3B).

Then, we investigated differentially expressed genes (DEGs) through single-cell RNA sequencing (scRNA-seq) to clarify *S. epidermidis*-induced genes related with the cellular entry of IAV in the nasal epithelium (Supplementary Fig. 2). As compared with NHNE cells, there were 5036 DEGs based on the cutoff criteria (adjusted *p* < 0.05, fold change ≥ or <1.5 and normalized data (log2) ≥ or <2.0) in *S. epidermidis*-inoculated NHNE cells at 1 dpi. We performed Gene Ontology (GO) enrichment analysis of scRNA-seq data by DAVID using cell lysates from the *S. epidermidis*-inoculated NHNE cells to confirm the effect of *S. epidermidis* in restricting host entry of IAV. The GO analysis showed that DEGs linked with biological processes were mainly enriched in “entry into host cells,” “entry into other organism involved in symbiotic interaction,” “entry into the host,” “viral entry into a host cell,” and “entry into host cells of other organism involved in symbiotic interaction” (Fig. 3C and Supplementary Table 1). We also performed GO enrichment analysis of scRNA-seq data by EnrichR using cell lysates from the *S. epidermidis*-inoculated NHNE cells and confirmed similar results about DEGs and GO categories with DAVID analysis (Supplementary Table 2).

Among 5036 DEGs, 19 genes were classified into the GO category linked with serine-type peptidase inhibitor activity. The scatterplot data of DEGs showed that both *Serpine1* and *Serpine2* genes increased the most among all genes linked with serine-type endopeptidase inhibitors in *S. epidermidis*-inoculated NHNE cells (Fig. 3D), and we confirmed increased normalized *Serpine1* and *Serpine2* expression in basal cells of nasal epithelium (Fig. 3E). The scRNA-seq data also revealed that the basal level of *Serpine1* (normalized data (log2): 3.723) was significantly higher than that of *Serpine2* (normalized data (log2): 1,216) in NHNE cells (Table 1). As a next step, we studied the expression kinetics of the human *Serpine1* and *Serpine2* genes as well as protein production in NHNE cells following inoculation with *S. epidermidis*. PCR results showed that *Serpine1* mRNA was significantly upregulated until 24 h, but the induction of the *Serpine2* gene was relatively lower in *S. epidermidis*-inoculated NHNE cells (Fig. 3F). Consistent with the induction of *Serpine1* gene expression, intracellular and secreted levels of Serpine1 protein were significantly elevated in *S. epidermidis*-inoculated NHNE cells, with the highest levels observed 24 h after inoculation (Fig. 3G, H). The heatmap of serine-type peptidase inhibitor transcript activity showed that the increase of *Serpine1* transcripts was the most significant in secretory-like (15.4-fold over control) and suprabasal cells (12.4-fold over control) (Fig. 3I and Supplementary Table 3).

**Table 1:** Differential expression gene (DEG) linked with serine-type peptidase inhibitors in *S. epidermidis*-inoculated NHNE cells.

ID	Gene symbol	Fold change	Normalized data (log2)		Annotation
		*S. epi* /Cont	Cont	*S. epi*	Seq names
5214	**SERPINE2**	40.804	1.216	6.566	NC_000002.12
12691	**SERPINE1**	17.155	3.723	7.824	NC_000007.14
5869	COL7A1	5.426	1.900	4.340	NC_000003.12
9772	SPINK1	5.147	1.889	4.252	NC_000005.10
9567	SPOCK1	5.062	2.492	4.832	NC_000005.10
23558	SERPINA1	4.863	0.423	2.705	NC_000014.9
26651	SERPINF2	4.134	0.357	2.405	NC_000017.11
11097	CD109	3.635	3.441	5.303	NC_000006.12
20307	A2ML1	3.348	0.280	2.023	NC_000012.12
23566	SERPINA3	2.633	4.401	5.797	NC_000014.9
29022	SERPINB7	2.482	3.985	5.296	NC_000018.10
29016	SERPINB5	2.367	5.555	6.798	NC_000018.10
29724	PI3	1.949	7.018	7.981	NC_000020.11
12562	TFPI2	1.843	3.957	4.839	NC_000007.14
33134	APP	1.731	6.742	7.534	NC_000021.9
3546	CRIM1	1.662	3.576	4.309	NC_000002.12
4861	TFPI	0.656	3.849	3.241	NC_000002.12
29023	SERPINB2	0.628	5.840	5.169	NC_000018.10
30716	CPAMD8	0.339	2.489	0.929	NC_000019.10

*Cont* control, NHNE cells without *S. epidermidis* inoculation, *S epi* *S. epidermidis*- inoculated NHNE cells.

To assess the effects of *S. epidermidis*-induced Serpine1 expression on the susceptibility of the nasal epithelium to IAV infection, NHNE cells were inoculated with *S. epidermidis* at an MOI of 0.25 and then infected with IAV 8 h after inoculation (MOI 1). Interestingly, PCR results revealed that *Serpine1* mRNA was also upregulated in IAV-infected NHNE cells (1.2 × 10^4^ ± 4.3 × 10^3^) at 1 dpi and *Serpine1* gene expression was also induced by *S. epidermidis* inoculation (1.1 × 10^4^ ± 3.1 × 10^3^). However, the *Serpine1* gene expression level was more highly induced in NHNE cells with *S. epidermidis* inoculation following IAV infection (3.9 × 10^4^ ± 5.2 × 10^3^; Fig. 3J). Subsequently, the IAV *PA* mRNA level was assessed in the cell lysate of *S. epidermidis*-inoculated NHNE cells following IAV infection depending on the neutralization of Serpine1 in NHNE cells. The increased IAV *PA* mRNA level (1.3 × 10^3^) was completely reduced in the cell lysate of IAV-infected NHNE cells with *S. epidermidis* inoculation (1.8 × 10^2^), but the cell lysate of IAV-infected NHNE cells treated with Serpine1 neutralizing antibody exhibited increased IAV mRNA level (2.4 × 10^2^) again regardless of *S. epidermidis* inoculation (Fig. 3K). Western blot analysis similarly revealed that IAV NP level was not reduced in IAV-infected NHNE cells that were treated with Serpine1 neutralizing antibody before *S. epidermidis* inoculation (Fig. 3L). As a next step, Serpine1 blocking peptide was inoculated into the nasal cavity of B6 mice (2.5 ug/5 ul) at 1 day prior to antibiotics treatment and then, *S. epidermidis* was inoculated with inoculated to the mice two days before IAV (2130 pfu/30 μl, PBS) infection. Both IAV *PA* mRNA and NP levels were assessed in the nasal mucosa of *S. epidermidis*-inoculated B6 mice following IAV infection depending on the neutralization of Serpine1 (Supplementary Fig. 3a). The increased IAV *PA* mRNA level (2.7 × 10^3^) was completely reduced in the nasal mucosa of IAV-infected B6 mice with *S. epidermidis* inoculation (3.2 × 10^2^), but the IAV-infected mice treated with Serpine1 neutralizing antibody exhibited increased IAV mRNA level (2.8 × 10^3^) again regardless of *S. epidermidis* inoculation (Supplementary Fig. 3b). Western blot analysis similarly revealed that IAV NP level was not reduced in the nasal mucosa of IAV-infected mice that were treated with Serpine1 neutralizing antibody before *S. epidermidis* inoculation (Supplementary Fig. 3c).

These findings indicate that nasal commensal *S. epidermidis*-regulated antiviral immune responses might be specially mediated by serine protease inhibitor in the nasal epithelium and that *S. epidermidis*-induced Serpine1 production might be involved in disturbing cellular entry of IAV in nasal epithelium.

### *S. epidermidis* induced Serpine1 expression in NHNE cells via interactions with an increase in urokinase plasminogen activator (uPA)

At last, we examined how *S. epidermidis* induced Serpine1 expression in the nasal epithelium and whether *S. epidermidis*-induced Serpine1 might be involved in the decrease of host proteases in NHNE cells.

**Fig. 4:**
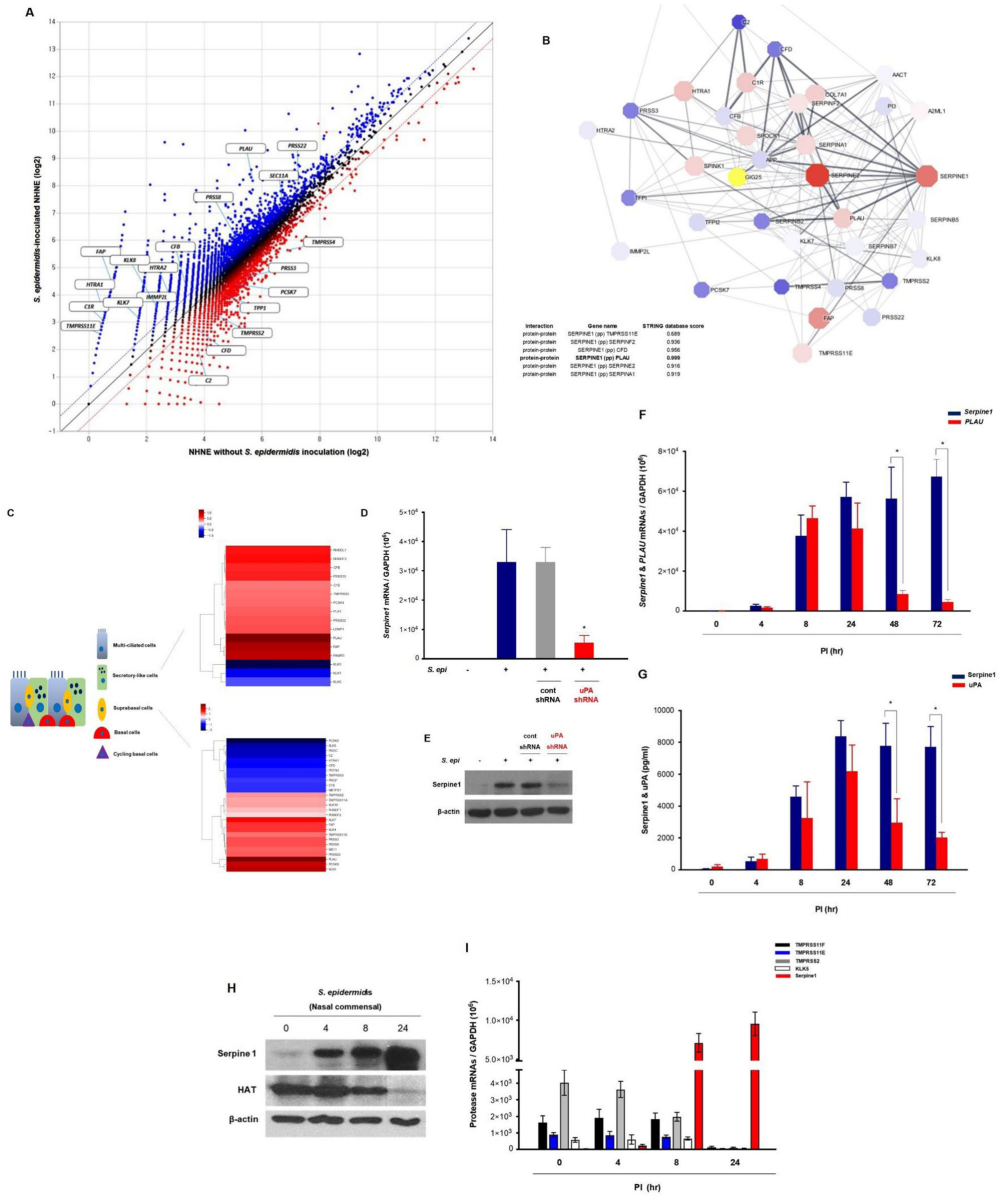
*Staphylococcus epidermidis*-induced Serpine1 expression was regulated by increase of uPA production and suppressed the activity of intracellular serine protease. **A** Scatterplots indicating enriched genes related with serine-type peptidase. **B** PPI network of DEGs linked with serine-type peptidase indicates the protein*–*protein interaction with Serpine1 and Serpine2 (PP protein*–*protein). **C** Heatmap depicting the expression levels of genes related with serine-type peptidase inhibitor activity differentially expressed in *S. epidermidis–*inoculated NHNE cells depending on a cellular subset. **D** Serpine1 mRNA and **E** protein levels were monitored by real-time PCR and western blot analysis following *S. epidermidis* inoculation at 1 dpi with transfection with cont shRNA and uPA shRNA. **F** *Serpine1* (blue bar) and *PLAU* mRNAs (red bar) were compared in NHNE cells after *S. epidermidis* inoculation. **G** ELISA results showed the secreted protein levels of Serpine1 (blue bar) and uPA (red bar) in *S. epidermidis*-inoculated NHNE cells. **H** The intracellular protein levels of HAT and Serpine1 were measured in the cell lysate of *S. epidermidis–*inoculated NHNE cells at 4, 8, and 24 h using western blot analysis. **I** The mRNA levels of proteases, such as TMPRSS2, TMPRSS11E, TMPRSS11F, and KLK5, which were targeted by Serpine1, were measured using the cell lysate of *S. epidermidis*-inoculated NHNE cells at 4, 8, and 24 h. Western blot results are representative of five independent experiments. Real-time PCR and plaque assay results are presented as mean ± SD values from five independent experiments. *p < 0.05 vs. mock-infected NHNE cells.

Among 5036 DEGs, 20 genes were classified into the GO category linked with serine-type peptidase activity, and the scatterplot data of DEGs showed that *PLAU* (urokinase plasminogen activator (uPA)), *HtrA serine peptidase1*, and *TMPRSS11E* were more significantly elevated in *S. epidermidis*-inoculated NHNE cells 24 h after inoculation (Fig. 4A). Our data also showed that *PLAU* gene expression was more highly induced among the DEGs linked with serine-type peptidases in *S. epidermidis*-inoculated NHNE cells (Table 2). A protein–protein interaction network of 39 DEGs linked with serine-type peptidase and serine-type peptidase inhibitors was constructed using the STRING online database and visualized by Cytoscape. Connectivity MAP analysis revealed the more characteristic protein–protein interaction (PPI) between *Serpine1* and *PLAU* (STRING database score: 0.999), and these two genes showed a more significant influence on maintaining the stability of the PPI network (Fig. 4B). The heatmap data revealed that the induction of *PLAU* gene expression was more significant in secretory-like and suprabasal cells as was also true with the expression of *Serpine1* (Fig. 4C). Next, NHNE cells were transfected with scrambled short hairpin (sh) RNA (cont shRNA) and uPA shRNA to suppress a transient expression of *PLAU* in NHNE cells before *S. epidermidis* inoculation. Interestingly, an increase in the *Serpine1* mRNA level in *S. epidermidis*-inoculated NHNE cells (3.2 × 10^4^) with the transfection of cont shRNA was significantly attenuated in NHNE cells with transfection of uPA shRNA (4.8 × 10^3^) before *S. epidermidis* inoculation (Fig. 4D). Western blot results revealed that *S. epidermidis*-induced protein levels of Serpine1 were also reduced in NHNE cells with transfection of uPA shRNA (Fig. 4E). We also found that IAV *PA* mRNA levels increased again in IAV-infected NHNE cells with transfection of uPA shRNA despite inoculation of *S. epidermidis* (Supplementary Fig. 4). These findings suggest that the nasal commensal *S. epidermidis* induces Serpine1 production via an increase of uPA in the nasal epithelium and that a sequential induction of uPA and Serpine1 might be related with inhibition of cellular entry of IAV in nasal epithelium.

**Table 2:** Differential expression gene (DEG) linked with serine-type peptidase in *S. epidermidis*-inoculated NHNE cells.

ID	Gene symbol	Fold change	Normalized data (log2)		Annotation
		*S. epi* /Cont	Cont	*S. epi*	Seq names
4659	FAP	11.843	0.823	4.389	NC_000002.12
19995	HTRA1	6.523	0.552	3.257	NC_000010.11
20262	C1R	5.882	0.505	3.062	NC_000012.12
19503	**PLAU**	5.627	5.676	8.168	NC_000010.11
7652	TMPRSS11E	4.505	0.391	2.563	NC_000004.12
31697	KLK7	2.668	1.728	3.143	NC_000019.10
31698	KLK8	2.308	2.349	3.555	NC_000019.10
12793	IMMP2L	2.308	2.349	3.555	NC_000007.14
3923	HTRA2	2.297	2.789	3.989	NC_000002.12
25798	PRSS8	1.933	5.116	6.067	NC_000016.10
10665	CFB	1.929	3.367	4.315	NC_000006.12
25259	PRSS22	1.761	7.264	8.080	NC_000016.10
24851	SEC11A	1.666	6.694	7.431	NC_000015.10
18630	PCSK7	0.631	5.053	4.389	NC_000011.10
17217	TPP1	0.599	4.715	3.975	NC_000011.10
15966	PRSS3	0.598	5.383	4.641	NC_000009.12
33346	TMPRSS2	0.595	4.147	3.399	NC_000021.9
30091	CFD	0.564	3.651	2.825	NC_000019.10
18646	TMPRSS4	0.418	6.891	5.632	NC_000011.10
10661	C2	0.292	3.777	1.999	NC_000006.12

*Cont* control, NHNE cells without *S. epidermidis* inoculation, *S epi* *S. epidermidis*- inoculated NHNE cells.

It has been reported that *PLAU* might be a major target of Serpine1 in the inhibitory mechanism of IAV infection in the respiratory epithelium^8^. Both *PLAU* mRNA and secreted protein levels of uPA gradually decreased in the cell lysate and supernatant of *S. epidermidis*-inoculated NHNE cells from 24 h after inoculation (Fig. 4F, G). *S. epidermidis* reduced the expression of other serine proteases identified as established targets of Serpine1 in the respiratory epithelium; notably, human airway trypsin (HAT) decreased immediately in the cell lysate of NHNE cells after *S. epidermidis* inoculation (Fig. 4H). We also found that *transmembrane protease serine (TMPRSS)2*, *TMPRSS11E*, *TMPRSS11F*, and *KLK5*, expression levels were gradually reduced in NHNE cells after *S. epidermidis* inoculation (Fig. 4I). In contrast, we were able to detect the gradual increase in the *Serpine1* gene and protein expression in NHNE cells until 72 h after *S. epidermidis* inoculation. These data showed that *S. epidermidis*-induced Serpine1 expression might be correlated with the reduction of airway proteases needed for IAV cellular entry to the nasal epithelium.

## Discussion

Our study revealed that *S. epidermidis*, which is abundant in healthy human nasal mucus, might be significantly associated with the induction of the serine protease inhibitor Serpine1 in the nasal epithelium and could restrict the spread of IAV to the lungs. Following inoculation of *S. epidermidis*, one of the serine proteases’ uPA induction stimulated Serpine1 production in the nasal epithelium and an increase in Serpine1 production reduced airway serine proteases, thereby skewing the protease–protease inhibitor balance of the nasal mucosa in favor of the host. This study highlights Serpine1 as a nasal commensal-regulated antiviral immune component in the healthy nasal mucosa and presents the role played by *S. epidermidis*-induced Serpine1 against the cellular invasion of IAV in the nasal epithelium.

The respiratory mucosa is the first target organ for environmental pathogens, and recent works have highlighted the critical role of the respiratory mucosa as a barrier for restricting invasion of the host by respiratory virus^20–24^. The compositional differences in the respiratory microbiome have drawn increased interest, and the importance of the respiratory microbiome, especially in immune protection, has been significantly recognized^18^. Human respiratory viruses first encounter host defense mechanisms in the nasal epithelium, and host protection can be conferred by a specialized innate immune system of the nasal epithelium capable of combating invasion by respiratory viruses^25,26^. There is also growing evidence that a microbiome community resides in the human nasal mucus and exhibits the potential for antiviral immunity. Our previous study revealed that *S. epidermidis* is the most abundant commensal organism in the healthy human nasal mucus, and the presence of *S. epidermidis* strengthens the frontline antiviral immune defense response in the respiratory tract through the modulation of interferon-dependent innate immune mechanisms in the nasal mucosa^15^. The current study also demonstrated that IAV-caused lung infection was more serious when the nasal microbiome was eliminated and that *S. epidermidis* can contribute to boosting immune responses, thereby suppressing acute IAV lung infection. We determined that additional studies are needed to demonstrate how the nasal commensal *S. epidermidis* mediates antiviral immune mechanisms and to define the clear mechanism of host–bacterial commensalism to impede the spread of respiratory viruses. The colonies of *S. epidermidis* were not found in the lungs but only in the nasal mucosa after intranasal inoculation. Therefore, we estimate that the *S. epidermidis* might contribute to potentiate antiviral immune responses at the level of the nasal mucosa and disturb viral spread to the lungs of IAV-infected mice.

To enter the airway epithelial cells, influenza, parainfluenza, and coronaviruses depend on trypsin-like serine proteases of the human airways for maturation of the viral protein involved in cell membranes^5,6,27^. Among several respiratory viruses, the mechanism of intracellular invasion to the respiratory epithelium by airway proteases has been well uncovered for influenza viruses. IAV maturation involves the cleavages of its surface glycoprotein hemagglutinin (HA) and cleavage by serine proteases is required for the cellular invasion or spread of IAV^7,8^. Therefore, HA cleavage has been proposed as a target for antiviral therapy and the mediators that could induce anti-protease activity can be used as a new therapeutic option against IAV infection in the respiratory tract.

An interesting point we noticed among the results of this study is that the viral titer was significantly higher in BAL fluid than in NAL fluid from IAV-infected mice. However, when comparing the viral titer in mice inoculated with *S. epidermidis* prior to IAV infection, a relatively large number of IAV particles was observed in NAL fluid and the viral titer was completely reduced in BAL fluid in response to inoculation of *S. epidermidis*, even though *S. epidermidis* reduced IAV replication in both the nasal mucosa and lung tissue. We assume that human nasal commensal *S. epidermidis* inhibited the intracellular invasion of IAV in the nasal epithelium and the mechanism by which IAV enters the nasal epithelium might be disturbed in the presence of *S. epidermidis*, which resulted in less spread of IAV from the nasal epithelium to the lungs of IAV-infected mice. Therefore, whether the nasal commensal *S. epidermidis* allowed the baseline serine protease inhibitor levels to be boosted and inhibited IAV replication through the induction of serine protease inhibitors in the nasal epithelium is our active inquiry and we extended our research through RNA-seq data and in vitro study using cultured human nasal epithelial cells.

Our scRNA-seq findings exhibited that the RNA levels of the serine protease inhibitor *Serpine1* were significantly induced in the cell lysate of *S. epidermidis*-inoculated NHNE cells and *Serpine1* induction was present more dominantly in secretory-like and suprabasal cells in the nasal epithelium. This observation was supported by in vitro studies revealing that the human nasal commensal *S. epidermidis* induced *Serpine1* gene expression and proteins in the cell lysate and supernatant of NHNE cells. Previously, it has been reported that Serpine1 inhibits multiple serine proteases with varying efficiencies, and HAT, TMPRSS families, and uPA-which are involved in extracellular IAV glycoprotein cleavage-are also targets of Serpine1 in the airway^5,8^. We found that *S, epidermidis*-induced Serpine1 expression showed an inverse correlation with the expression of airway proteases such as HAT, TMPRSS11E, TMPRSS11F, TMPRSS2, and KLK5, and the neutralization of Serpine1 activity exhibited higher IAV replication in *S. epidermidis*-inoculated nasal epithelial cells. These findings suggest that nasal commensal *S. epidermidis* has a distinctive antiviral strategy against IAV that is involved in the induction of serine protease inhibitors and turns the host nasal epithelium into an airway protease-deficient environment.

Serine protease inhibitors may be considered an emerging broad therapeutic group against multiple respiratory viruses that rely on these enzymes for their replication, and the clinical evidence are already available to suppress influenza viral replication through the inhibition of serine proteases^28,29^. We also propose that targeting serine proteases has a strong impact in reducing the spread of IAV from the nasal epithelium, thus identifying *S. epidermidis*-induced Serpine1 as an influence on the development of antiviral biologics to suppress IAV-caused lung infections. Therefore, our additional research focused on verifying how *S. epidermidis* induced Serpine1 expression in the nasal epithelium, and our data highlight that the upregulation of Serpine1 expression by *S. epidermidis* during IAV infection depends upon uPA induction in NHNE cells. Although the expression of other host proteases decreased in *S. epidermidis*-inoculated NHNE cells, both mRNA and protein levels of uPA were significantly increased in response to *S. epidermidis*. In this regard, it will be of interest to determine whether the induction of uPA by the nasal commensal *S. epidermidis* is linked with the change in cellular environment to Serpine1 induction. Our scRNA-seq data provided evidence that a close protein–protein interaction between uPA and Serpine1 was observable in *S epidermidis*-inoculated NHNE cells. Furthermore, the significant increase in Serpine1 expression in *S epidermidis*-inoculated NHNE cells was not observed with knockdown of the transient expression of uPA. The expression of uPA increased by *S. epidermidis* could be involved in Serpine1 induction, but, finally, uPA expression began to decrease from 24 h onward after inoculation with *S. epidermidis*. The current findings indicate that *S. epidermidis*-induced uPA is responsible for the sequential increase in Serpine1 expression, which results in reduced serine proteases in the nasal epithelium and thus may impede IAV spread to the respiratory tract.

Here, we described that the nasal commensal S*. epidermidis* suppresses IAV invasion to the nasal epithelium through uPA-dependent Serpine1 production and influences the serine protease-deficient cellular environment. This intimate association of *S. epidermidis* and the amplification of serine protease inhibitors could potentially benefit the host respiratory tract and prevent IAV spread to the lungs through the downregulation of airway proteases at the level of the nasal mucosa.

## Methods

### Ethics statement

Participation in this study was voluntary, with written informed consent obtained from all human subjects prior to enrollment. The institutional review board (IRB) of the Seoul National University College of Medicine approved the protocol for this study (IRB #C2012248 [943]). In vivo experiments with C57BL/6 J mice were carried out according to guidelines approved by the IRB of the Seoul National University College of Medicine (IACUC #2016–0093).

### Nasal mucus microbiome characterization

The mucus from the middle turbinate of healthy volunteers was collected individually using sterile 3 M Quick swabs (3 M Microbiology Products, St. Paul, MN, USA) from four subjects using a rigid 0-degree endoscope in an operating room. The swabs with mucus were fixed in a fixative solution and transported immediately to the laboratory for identification and subsequent microbial analysis. For bacterial colony isolation, the mucus was placed on lysogeny broth plates. After two days of incubation, bacterial colonies were obtained from the lysogeny broth plates and the species of each colony were identified using GS-FLX 454 pyrosequencing by 16 S ribosomal RNA gene amplification^15^. *Staphylococcus epidermidis* strains (N1–N4) were isolated from four individuals, and all four strains isolated were used in this study (Supplementary Fig. 5).

### Viruses and reagents

Influenza A virus strain A/Wilson–Smith/1933 H1N1 (IAV A/WS/33; American Type Culture Collection, Manassas, VA, USA) was used in this study to induce acute viral lung infection. Virus stocks were grown in Madin–Darby canine kidney (MDCK) cells in a viral growth medium according to a standard procedure^30^. Briefly, after 48 h of incubation at 37 °C, the supernatants were harvested and spun by centrifugation at 5000 rpm for 30 min to remove cellular debris. Virus stocks were titrated on MDCK cells using a tissue culture infectious dose assay and stored at −80 °C. Control shRNA (cat # sc-108080) and uPA shRNA (cat # sc-36779) lentiviral particles were purchased from Santa Cruz Biotechnology (Dallas, Texas USA).

### Cell culture

Passage-2 NHNE cells (1 × 10^5^ cells/culture) were seeded in 0.25 mL of culture medium on Transwell clear culture inserts (24.5 mm, with a 0.45-mm pore size; Costar Co., Cambridge, MA, USA). Cells were cultured in a 1:1 mixture of basal epithelial growth medium and Dulbecco’s modified Eagle medium containing the previously described supplements. Cultures were grown while submerged for the first 9 days. The culture medium was changed on day 1, then on every other day thereafter. An air–liquid interface (ALI) was created on day 9 by removing the apical medium and feeding the cultures from the basal compartment only. The culture medium was changed daily after the establishment of the ALI. An antifungal agent, fungizone (1 ml/1,000 mL media; Life Technologies, Grand Island, NY, USA) was added after filtering the media. All experiments described here employed cultured nasal epithelial cells at 14 days after the creation of the ALI^31^.

### Real-time PCR

NHNE cells were infected with WS/33 (H1N1) for 10 and 30 min; 8, 2, and 8 h; and 1, 2, and 3 days, respectively, and total RNA was isolated using TRIzol reagent (Life Technology, Seoul, Korea). Complementary DNA (cDNA) was synthesized from 3 μg of RNA with random hexamer primers (Perkin Elmer Life Sciences, Waltham, MA, USA) and Moloney murine leukemia virus reverse transcriptase (Roche Applied Science, Indianapolis, IN, USA). Amplification was performed using TaqMan Universal PCR Master Mix (PE Biosystems, Foster City, CA, USA) according to the manufacturer’s protocol. Briefly, amplification reactions had a total volume of 12 μL and contained 2 μL of cDNA (reverse transcription mixture), oligonucleotide primers (final concentration of 800 nM), and TaqMan hybridization probe (200 nM). Real-time PCR probes were labeled at the 5′ end with carboxyfluorescein (FAM) and the 3′ end with the quencher carboxytetramethylrhodamine (TAMRA). To quantify the cellular viral level and host gene expression, cellular RNA was used to generate cDNA. The IAV level was monitored by performing quantitative PCR of the *PA* gene (segment 3) with forward and reverse primers and the probes 5′-ggccgactacactctcgatga-3′, 5′-tgtcttatggtgaatagcctggttt-3′, and 5′-agcagggctaggatc-3′, respectively. Primers for human or mouse Serpine1 (assay ID Hs00167155_m1, Mm00435858_m1), Serpine2 (assay ID Hs00299953_m1, Mm00436753_m1), TMPRSS11E (assay ID Hs01070171_m1, Mm01212186_m1), TMPRSS11F (assay ID Hs01592083_m1, Mm00812591_m1), TMPRSS2 (assay ID Hs01122322_m1, Mm00443687_m1), and KLK5 (assay ID Hs01548153_m1, Mm01203811_m1) were purchased from Applied Biosystems (Foster City, CA, USA). Real-time PCR was performed using the PE Biosystems ABI PRISM^®^ 7700 sequence detection system. Thermocycling parameters were as follows: 50 °C for 2 min, 95 °C for 10 min, then 40 cycles of 95 °C for 15 sec and 60 °C for 1 min. Target mRNA levels were quantified using target-specific primer and probe sets for IAV WS/33 (H1N1), Serpine1, Serpine2, TMPRSS11E, TMPRSS11F, TMPRSS2, and KLK5. All PCR assays were quantitative and used plasmids containing the target gene sequences as standards. All reactions were performed in triplicate, and all real-time PCR data were normalized to the level of glyceraldehyde phosphate dehydrogenase (GAPDH, 1 × 10^6^ copies) to correct for variations between samples.

### Quantification of secreted proteins

Secreted human urokinase plasminogen activator and Serpine1 were quantified using human urokinase plasminogen activator (DY1310) and human Serpine1 (DY1786) DuoSet enzyme-linked immunosorbent assay (ELISA) kits (R&D Systems, Minneapolis, MN, USA), respectively. The working range of the assays was 62.5 to 4000 pg/mL.

### Viral titer determination

Viral titers were determined using a plaque assay. Virus samples were serially diluted with PBS. Confluent monolayers of MDCK cells in twelve-well plates were washed twice with PBS, then infected in duplicate with 250 μL/well dilution of each virus. The plates were incubated at 37 °C for 45 min to facilitate virus adsorption. Following adsorption, a 1% agarose overlay in complete MEM supplemented with TPCK trypsin (1 μg/mL) and 1% fetal bovine serum was applied. The plates were then incubated at 37 °C, and cells were fixed with 10% formalin at 2 dpi.

### Western blot analysis

The IAV NP, HAT, and Serpine1 protein levels were assessed using western blot analysis. IAV NP antibody (molecular weight 61 kDa, primary antibody 1:500) was purchased from Fitzerlard (North action, MA, USA, Cat #10R-2121). Human HAT antibody (molecular weight 46 kDa, cat# MAB2695-SP, primary antibody 1:500), anti-β-actin antibody (molecular weight 42 kDa, primary antibody 1:500), and human Serpine1 neutralizing antibody (cat #AF-1786) were purchased from the R&D system (Minneapolis, MN, USA). In addition, a human Serpine1 antibody (molecular weight 45 kDa, primary antibody 1:500) was purchased from Abcam (Waltham, MA, USA, cat #ab66705) and mouse Serpine1 blocking peptide from MyBioSource (San Diego, CA, USA, cat #MBS8309331). San Diego, CA, USA). The NHNE cells were lysed with 2× lysis buffer (250 mM Tris-Cl, pH of 6.5, 2% SDS, 4% β-mercaptoethanol, 0.02% bromophenol blue, and 10% glycerol). Cell lysate (30 μg of protein) was electrophoresed in 10% SDS gels and transferred to polyvinylidene difluoride membranes in Tris-buffered saline (TBS) (50 mM Tris-Cl, pH of 7.5, 150 mM of NaCl) for 1 h at room temperature. The membrane was incubated overnight with primary antibody (1:500) in Tween-Tris–buffered saline (TTBS) (0.5% Tween-20 in TBS). After washing with TTBS, the blot was incubated for one hour at room temperature with secondary anti-rabbit or anti-mouse antibody (1:1,000, Cell Signaling Technologies, Danvers, MA, USA) in TTBS and was visualized using an ECL system (Amersham, Little Chalfont, England). The full uncropped scans of the western blots included in the results of the present study are provided in supplementary Fig. 6.

### Murine infection model

Male C57BL/6 J (B6) mice (Orientalbio, Seoul, Korea) aged seven weeks (and weighing 19–23 g) were used as wild-type (WT) mice. The B6 mice used in the study were like other commercially available strains of inbred mice. For inoculation of the nasal microbiome, *S. epidermidis* (3.2 × 10^6^ CFUs in 30 μL of PBS) were delivered into the nasal cavity of WT mice and, for viral infection, IAV (WS/33, H1N1; 2130 pfu in 30 μL of PBS) was inoculated into WT mice by intranasal delivery. After euthanizing the mice, BAL fluid was obtained from the lungs by lavaging with 1,000 μL of 0.5-mM ethylene diamine tetraacetic acid in PBS following cannulation of the trachea. The collected BAL fluid was used for ELISA for measuring secreted protein levels and plaque assay to determine the viral titer. Mouse lung tissue was also harvested for real-time PCR, microarray, and immunohistochemistry analyses.

### Immunohistochemistry and histologic analysis

Lung tissue was fixed in 10% (vol/vol) neutral buffered formalin and embedded in paraffin. Paraffin-embedded tissue slices were stained with hematoxylin and eosin (H&E) or periodic acid–Schiff (PAS) solution (Sigma-Aldrich, St. Louis, MO, USA). Histopathologic analysis of inflammatory cells in H&E-stained lung sections was performed in a blinded fashion using a semi-quantitative scoring system as described previously^32^. Lung sections from at least five mice were examined. Briefly, peribronchiolar inflammation was scored as follows: zero points, normal; one point, a few cells; two points, a ring of inflammatory cells one layer deep; three points, a ring of inflammatory cells two to four cells deep; or four points, a ring of inflammatory cells more than four cells deep (maximum possible score = 8 points). The histological score for PBS/PBS control mouse lung tissue was always zero points. At least six separate areas from similar sections within a single mouse were assessed, and at least five mice were assessed. PMNs were counted by an examiner who was blinded to the experimental group and expressed as the number of cells per high-power field.

### Single-cell RNA sequencing (scRNA-seq)

Library construction was performed using 10 × chromium single-cell 3’ version 3.1 reagent kits (10 × Genomics, Pleasanton, CA, USA). Samples were sequenced using the NovaSeq 6000 platform (Illumina, San Diego, CA, USA), and preliminary sequencing results were converted to FASTQ files using the Cell Ranger pipeline (10× Genomics). We followed the 10× Genomics standard sequence protocol by trimming the barcode and unique molecular identifier end to 26 bp and the mRNA end to 98 bp, respectively. Then, the FASTQ files were aligned to the human reference genome (GRCh38). Subsequently, we applied Cell Ranger for preliminary data analysis and generated a file that contained a barcode table, a gene table, and a gene-expression matrix. We used WinSeurat version 2.1 (Ebiogen Inc., Seoul, Korea), based on Seurat version 3, for quality control, analysis, and exploration of scRNA-seq data^33,34^. Data mining and graphic visualization were performed using ExDEGA (Ebiogen Inc).

The original scRNA-seq data of the NHNE cells with *S. epidermidis* inoculation are available at https://www.ncbi.nlm.nih.gov/geo/query/acc.cgi?acc=GSE167509.

### Statistical analyses

For in vitro study, at least three independent experiments were performed with cultured cells from each donor, and the results are presented as mean ± standard deviation (SD) values of triplicate cultures. Differences between treatment groups were evaluated by repeated measure two-way analysis of variance (ANOVA) and two-sample *t*-test was used to analyze scRNA-seq data. We present the in vivo results of real-time PCR, plaque assays, and ELISA as mean ± SD values from five individual mice and statistical analyses were performed by Mann–Whitney *U*-test. The survival rate of IAV-infected or *S. epidermidis*-inoculated mice were analyzed by Kaplan–Meier with log-rank test. GraphPad Prism (version 8; GraphPad Software, La Jolla, CA, USA) were used for these statistical analyses and differences were considered significant at *p* value < 0.05.

### Reporting Summary

Further information on research design is available in the Nature Research Reporting Summary linked to this article.

## Supplementary information

Reporting Summary Checklist

New SI requested by ME

Supplementary information

## Data Availability

The authors can confirm that all relevant data are included in the manuscript and its supplementary information files. The original scRNA-seq data are available at https://www.ncbi.nlm.nih.gov/geo/query/acc.cgi?acc=GSE167509.

## References

[1] Garcia-Sastre A, Biron CA (2006). Type 1 interferons and the virus-host relationship: a lesson in detente. Science.

[2] Cheon H (2013). IFNbeta-dependent increases in STAT1, STAT2, and IRF9 mediate resistance to viruses and DNA damage. EMBO J..

[3] Kim HJ (2013). Reactive oxygen species induce antiviral innate immune response through IFN-lambda regulation in human nasal epithelial cells. Am. J. Respir. Cell Mol. Biol..

[4] Galani IE (2017). Interferon-λ mediates non-redundant front-line antiviral protection against influenza virus infection without compromising host fitness. Immunity.

[5] Laporte M, Naesens L (2017). Airway proteases: an emerging drug target for influenza and other respiratory virus infections. Curr. Opin. Virol..

[6] Bertram S (2010). Novel insights into proteolytic cleavage of influenza virus hemagglutinin. Rev. Med. Virol..

[7] Watanabe T, Watanabe S, Kawaoka Y (2010). Cellular networks involved in the influenza virus life cycle. Cell Host Microbe.

[8] Dittmann M (2015). A serpin shapes the extracellular environment to prevent influenza A virus maturation. Cell.

[9] Kim S (2017). The superiority of IFN-λ as a therapeutic candidate to control acute influenza viral lung infection. Am. J. Respir. Cell Mol. Biol..

[10] Won J (2019). Inhaled delivery of Interferon-lambda restricts epithelial-derived Th2 inflammation in allergic asthma. Cytokine.

[11] Segal LN, Rom WN, Weiden MD (2014). Lung microbiome for clinicians. New discoveries about bugs in healthy and diseased lungs. Ann. Am. Thorac. Soc..

[12] Suzaki H, Watanabe S, Pawankar R (2013). Rhinosinusitis and asthma-microbiome and new perspectives. Curr. Opin. Allergy Clin. Immunol..

[13] Brestoff JR, Artis D (2013). Commensal bacteria at the interface of host metabolism and the immune system. Nat. Immunol..

[14] Liu Q (2010). Staphylococcus epidermidis contributes to healthy maturation of the nasal microbiome by stimulating antimicrobial peptide production. Cell Host Microbe.

[15] Kim HJ (2019). Nasal commensal Staphylococcus epidermidis enhances interferon-λ-dependent immunity against influenza virus. Microbiome.

[16] Sahin-Yilmaz A, Naclerio RM (2011). Anatomy and physiology of the upper airway. Proc. Am. Thorac. Soc..

[17] Basis CM (2014). The nasal cavity microbiota of healthy adults. Microbiome.

[18] Basis CM (2015). Analysis of the upper respiratory tract microbiotas as the source of the lung and gastric microbiotas in healthy individuals. MBio.

[19] Iwase T (2010). Staphylococcus epidermidis Esp inhibits Staphylococcus aureus biofilm formation and nasal colonization. Nature.

[20] Wies E (2013). Dephosphorylation of the RNA sensors RIG-I and MDA5 by the phosphatase PP1 is essential for innate immune signaling. Immunity.

[21] Slater L (2010). Co-ordinated role of TLR3, RIG-I and MDA5 in the innate response to rhinovirus in bronchial epithelium. PLoS Pathog..

[22] Allen IC (2009). The NLRP3 inflammasome mediates in vivo innate immunity to influenza A virus through recognition of viral RNA. Immunity.

[23] Iwasaki A, Pillai PS (2014). Innate immunity to influenza virus infection. Nat. Rev. Immunol..

[24] Pang IK, Iwasaki A (2011). Inflammasomes as mediators of immunity against influenza virus. Trends Immunol..

[25] Tarabichi Y (2015). The administration of intranasal live attenuated influenza vaccine induces changes in the nasal microbiota and nasal epithelium gene expression profiles. Microbiome.

[26] Beck JM, Young VB, Huffnagle GB (2012). The microbiome of the lung. Transl. Res..

[27] Chen J (1998). Structure of the hemagglutinin precursor cleavage site, a determinant of influenza pathogenicity and the origin of the labile conformation. Cell.

[28] Zhirnov OP, Klenk HD, Wright PF (2011). Aprotinin and similar protease inhibitors as drugs against influenza. Antivir. Res..

[29] Meyer D (2013). Identification of the first synthetic inhibitors of the type II transmembrane serine protease TMPRSS2 suitable for inhibition of influenza virus activation. Biochem J..

[30] An S (2018). Initial influenza virus replication can be limited in allergic asthma through rapid induction of type III interferons in respiratory epithelium. Front. Immunol..

[31] Park DY (2016). Alternative method for primary nasal epithelial cell culture using intranasal brushing and feasibility for the study of epithelial functions in allergic rhinitis. Allergy Asthma Immunol. Res..

[32] Koltsida O (2011). IL-28A (IFN-lambda2) modulates lung DC function to promote Th1 immune skewing and suppress allergic airway disease. EMBO Mol. Med..

[33] Butler A (2018). Integrating single-cell transcriptomic data across different conditions, technologies, and species. Nat. Biotechnol..

[34] Stuart T (2019). Comprehensive integration of single-cell data. Cell.

