# Supplementary material for: Nasal symbiont Staphylococcus epidermidis restricts the cellular entry of influenza virus into the nasal epithelium
Source: NPJ Biofilms Microbiomes. 2022 Apr 13;8:26. doi: 10.1038/s41522-022-00290-3 (PMC9007948; doi:10.1038/s41522-022-00290-3)
Supplement: Supplementary file 1 — Reporting Summary Checklist [file 41522_2022_290_MOESM1_ESM.pdf]

nature portfolio

Corresponding author(s): Hyun Jik KIm

Last updated by author(s): Jan 27, 2022

Reporting Summary

Nature Portfolio wishes to improve the reproducibility of the work that we publish. This form provides structure for consistency and transparency in reporting. For further information on Nature Portfolio policies, see our Editorial Policies and the Editorial Policy Checklist.

Statistics

For all statistical analyses, confirm that the following items are present in the figure legend, table legend, main text, or Methods section.

n/a	Confirmed	
☐	☒	The exact sample size (n) for each experimental group/condition, given as a discrete number and unit of measurement
☐	☒	A statement on whether measurements were taken from distinct samples or whether the same sample was measured repeatedly
☐	☒	The statistical test(s) used AND whether they are one- or two-sided<br>*Only common tests should be described solely by name; describe more complex techniques in the Methods section.*
☐	☒	A description of all covariates tested
☐	☒	A description of any assumptions or corrections, such as tests of normality and adjustment for multiple comparisons
☐	☒	A full description of the statistical parameters including central tendency (e.g. means) or other basic estimates (e.g. regression coefficient) AND variation (e.g. standard deviation) or associated estimates of uncertainty (e.g. confidence intervals)
☒	☐	For null hypothesis testing, the test statistic (e.g. F, t, r) with confidence intervals, effect sizes, degrees of freedom and P value noted<br>*Give P values as exact values whenever suitable.*
☒	☐	For Bayesian analysis, information on the choice of priors and Markov chain Monte Carlo settings
☒	☐	For hierarchical and complex designs, identification of the appropriate level for tests and full reporting of outcomes
☒	☐	Estimates of effect sizes (e.g. Cohen's d, Pearson's r), indicating how they were calculated

Our web collection on statistics for biologists contains articles on many of the points above.

Software and code

Policy information about availability of computer code

Data collection	WinSeurat Version 2.1 (Single cell RNA sequencing)
Data analysis	GraPad Prism (version 8)

For manuscripts utilizing custom algorithms or software that are central to the research but not yet described in published literature, software must be made available to editors and reviewers. We strongly encourage code deposition in a community repository (e.g. GitHub). See the Nature Portfolio guidelines for submitting code & software for further information.

Data

Policy information about availability of data

All manuscripts must include a data availability statement. This statement should provide the following information, where applicable:

- Accession codes, unique identifiers, or web links for publicly available datasets
- A description of any restrictions on data availability
- For clinical datasets or third party data, please ensure that the statement adheres to our policy

https://www.n cbi.nlmn. ih .gov/geo/ q uery/ acc.cgia?c c=GSE167509

Field-specific reporting

Please select the one below that is the best fit for your research. If you are not sure, read the appropriate sections before making your selection.

- [X] Life sciences
- [ ] Behavioural & social sciences
- [ ] Ecological, evolutionary & environmental sciences

For a reference copy of the document with all sections, see nature.com/documents/nr-reporting-summary-flat.pdf

Life sciences study design

All studies must disclose on these points even when the disclosure is negative.

Sample size	Fori n vitro study,a t leastt hree independente xperimentsw ere performedw ith culturedc ellsf rom eachd onor,a nd the resultsa re presented as meant standardd eviation(SD)v alueso ftriplicate cultures.D ifferencesb etweent reatmentg roupsw ere evaluatedb y repeatedm easure two-way analysiso f variance(ANOVAa) nd two samplet -test was usedt o analyzes cRNA-sedqa ta.W e presentt he in vivo resultso f real-time PCRp, laquea ssaysa, nd ELISAa s mean1 SD valuesf rom five individuaml icea nd statisticaal nalysesw ere performedb y Mann-WhitneyU test.
Data exclusions	N/A
Replication	N/A
Randomization	N/A
Blinding	N/A

Reporting for specific materials, systems and methods

We require information from authors about some types of materials, experimental systems and methods used in many studies. Here, indicate whether each material, system or method listed is relevant to your study. If you are not sure if a list item applies to your research, read the appropriate section before selecting a response.

Materials & experimental systems

n/a	Involved in the study	
[ ]	[X]	Antibodies
[X]	[ ]	Eukaryotic cell lines
[X]	[ ]	Palaeontology and archaeology
[ ]	[X]	Animals and other organisms
[X]	[ ]	Human research participants
[X]	[ ]	Clinical data
[X]	[ ]	Dual use research of concern

Methods

n/a	Involved in the study	
[X]	[ ]	ChIP-seq
[X]	[ ]	Flow cytometry
[X]	[ ]	MRI-based neuroimaging

Antibodies

Antibodies used	The IAVN P,H AT,a nd Serpinel proteinl evelsw ere assessedu singw esternb lot analysisI.A VN Pa ntibody(primarya ntibody1 :500) was purchasedfr om Fitzerlard(N ortha ction,M A, USA,C at# 10R-2121)H. umanH ATa ntibody(cat#M AB2595-SPp,r imarya ntibody 1:500),a nd humanS erpinel neutralizinga ntibodyw ere purchasedfr om R&Ds vstem(MinneapolisM, N, USA,c at #AF-1786)I.n addition,h umanS erpinel antibody(primarya ntibody1 :500)w as purchasedfr om Abcam(Waltham,l VA,U SA,c at #ab66705)a nd mouseS erpinel blockingp eptidef rom MvBiosource(S anD iego,C A,U SA,c at #M858309331).
Validation	N/A

Animals and other organisms

Policy information about studies involving animals; ARRIVE guidelines recommended for reporting animal research

Laboratory animals	C57BL/6
Wild animals	N/A
Field-collected samples	N/A
Ethics oversight	N/A

Note that full information on the approval of the study protocol must also be provided in the manuscript.
