# Supplementary material for: Nasal symbiont Staphylococcus epidermidis restricts the cellular entry of influenza virus into the nasal epithelium
Source: NPJ Biofilms Microbiomes. 2022 Apr 13;8:26. doi: 10.1038/s41522-022-00290-3 (PMC9007948; doi:10.1038/s41522-022-00290-3)
Supplement: Supplementary file 2 — New SI requested by ME [file 41522_2022_290_MOESM2_ESM.pdf]

Supplementary figure 1. Colony assay using NAL fluid of C57BL/6 mouse depending on intranasal antibiotics treatment. For bacterial colony isolation, the NAL fluid of B6 mouse was placed on lysogeny broth (LB) plates for 1 day. No bacterial colony was observed in NAL fluid from B6 mouse with intranasal antibiotic treatment.

NAL fluid

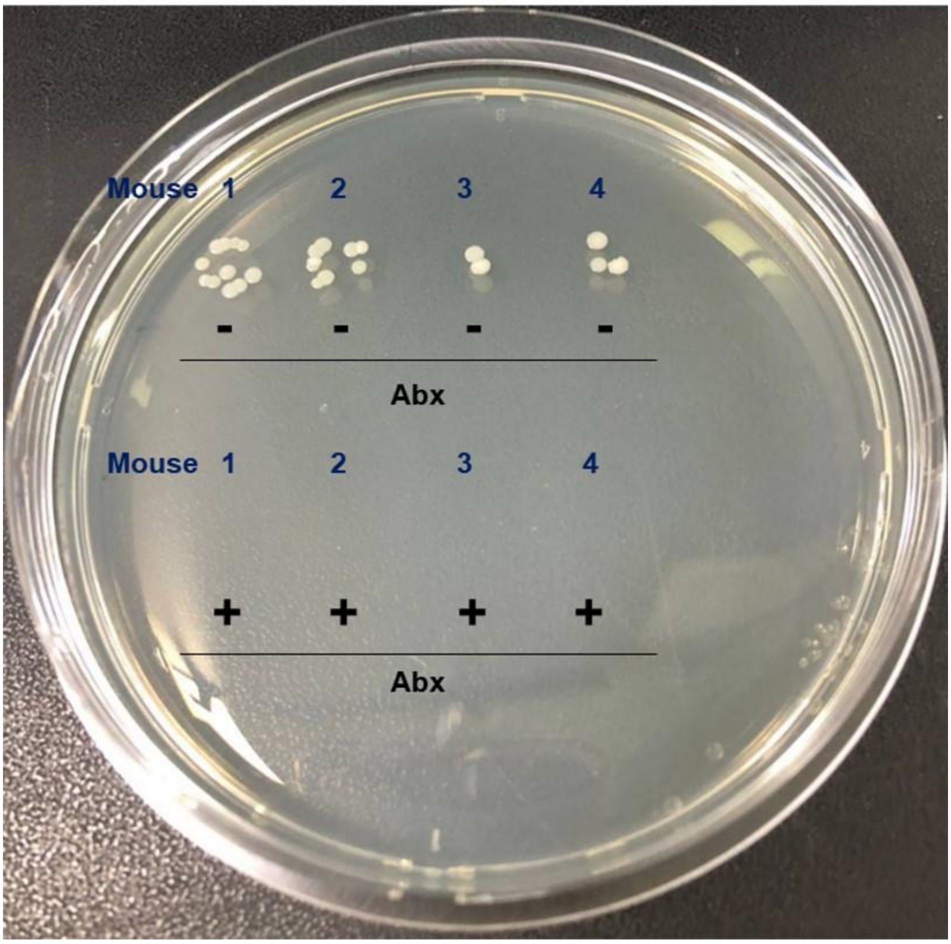

Supplementary figure 2. Schematic illustration depicts the sampling location of surgical nasal mucosa specimen (middle turbinate) for primary nasal epithelial culture and overview of the major cell types in the nasal epithelial cells for single cell RNA sequencing.

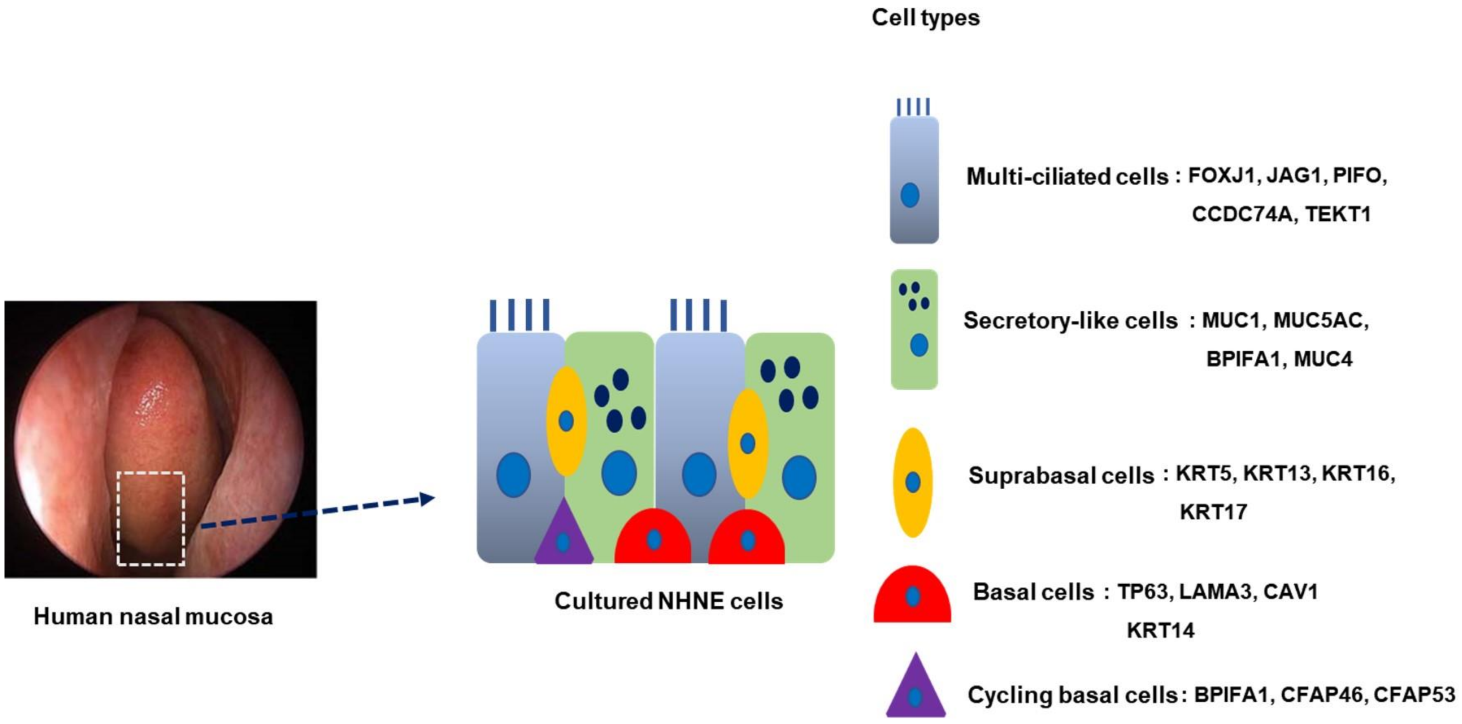

Supplementary figure 3. Serpine1 blocking peptide (2.5ug/ 5ul) was inoculated into the nasal cavity of B6 mice at 1 days prior to antibiotics treatment and then, *S. epidermidis* was inoculated with inoculated to the mice two days before IAV (2,130 pfu/30 μl, PBS) infection. (A) Serpine1 protein level was measured using western blot analysis. (B) IAV *PA* mRNA and (C) NP levels were assessed in the nasal mucosa of *S. epidermidis*-inoculated B6 mice following IAV infection depending on the neutralization of Serpine1.

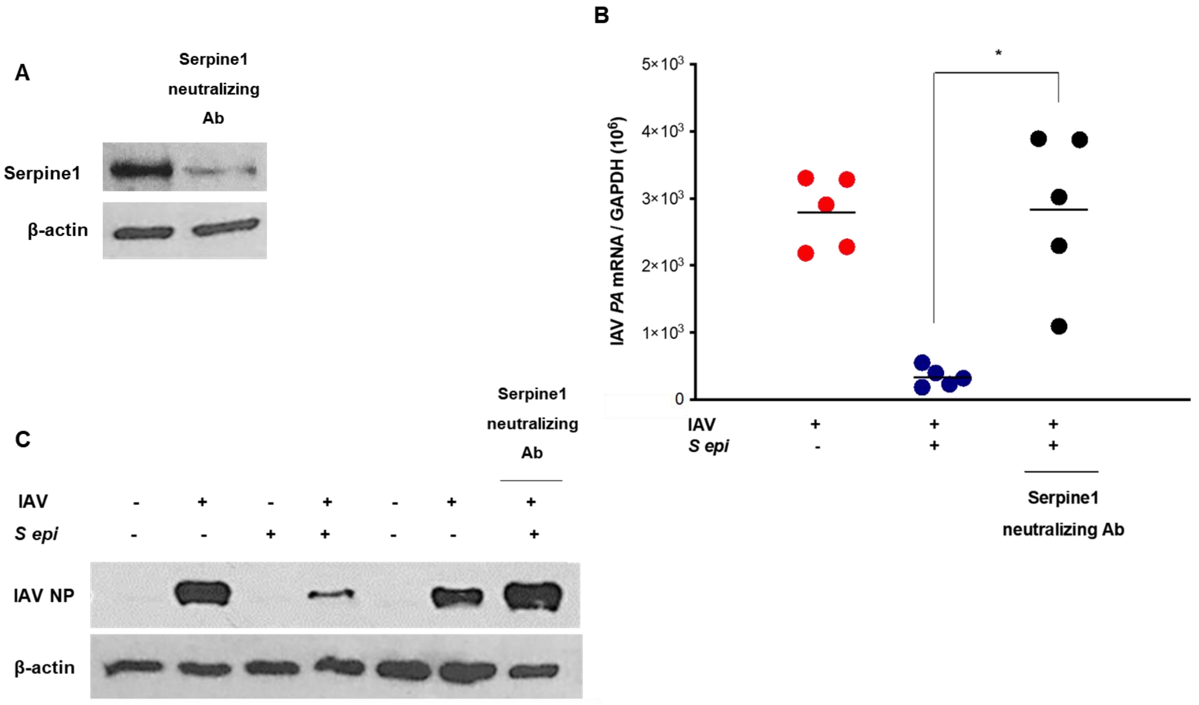

Supplementary figure 4. *Staphylococcus epidermidis*-induced uPA production was involved in IAV replication in NHNE cells. IAV *PA* mRNA levels were monitored by real-time PCR following *S. epidermidis* inoculation at 1 dpi with transfection with cont shRNA and uPA shRNA.

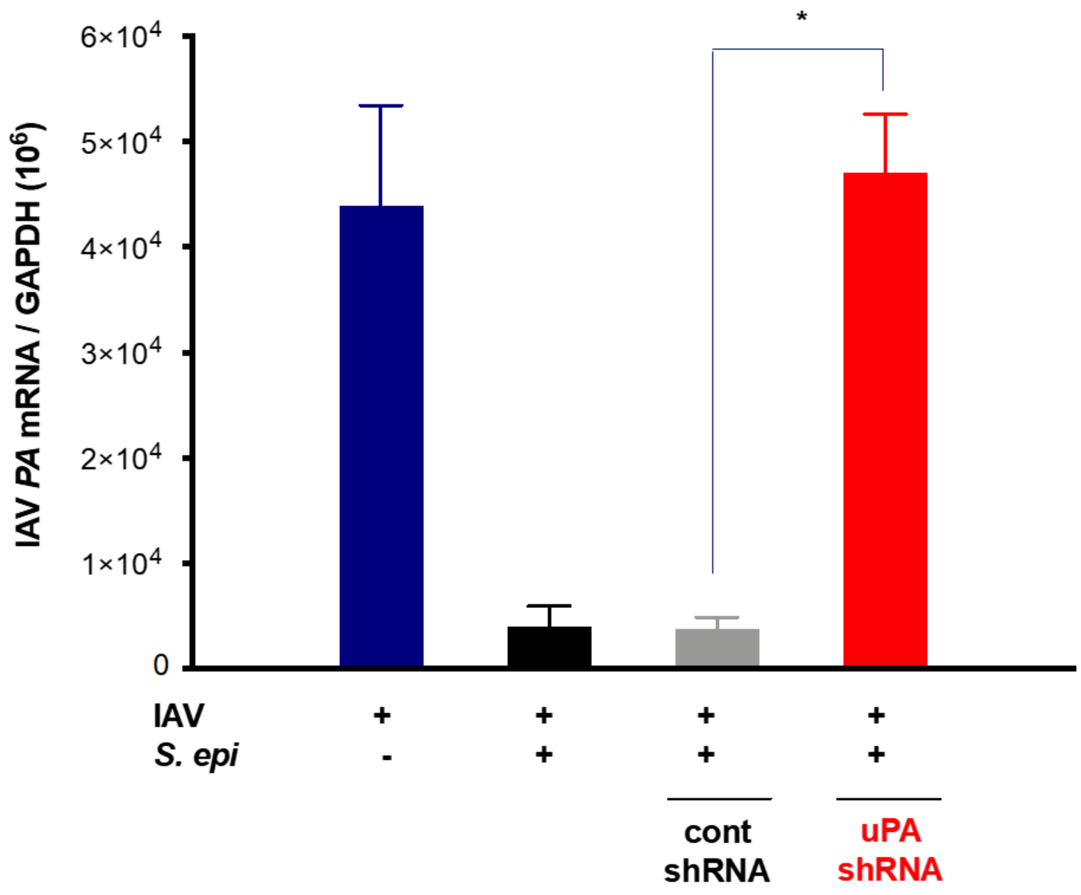

Supplementary figure 5. Schematic illustration of step-by-step experiments from *S. epidermidis* isolation to inoculation of NHNE cells. For bacterial colony isolation, nasal mucus of healthy subjects was obtained using a cotton swab and was used to inoculate Lysogeny Broth (LB) plates. After 2 days of incubation, bacterial colonies were obtained from the LB plates, *S. epidermidis* colonies were identified using GS-FLX 454 pyrosequencing by 16S rRNA gene amplification, and *S. epidermidis* was used to inoculate NHNE cells (MOI 0.25).

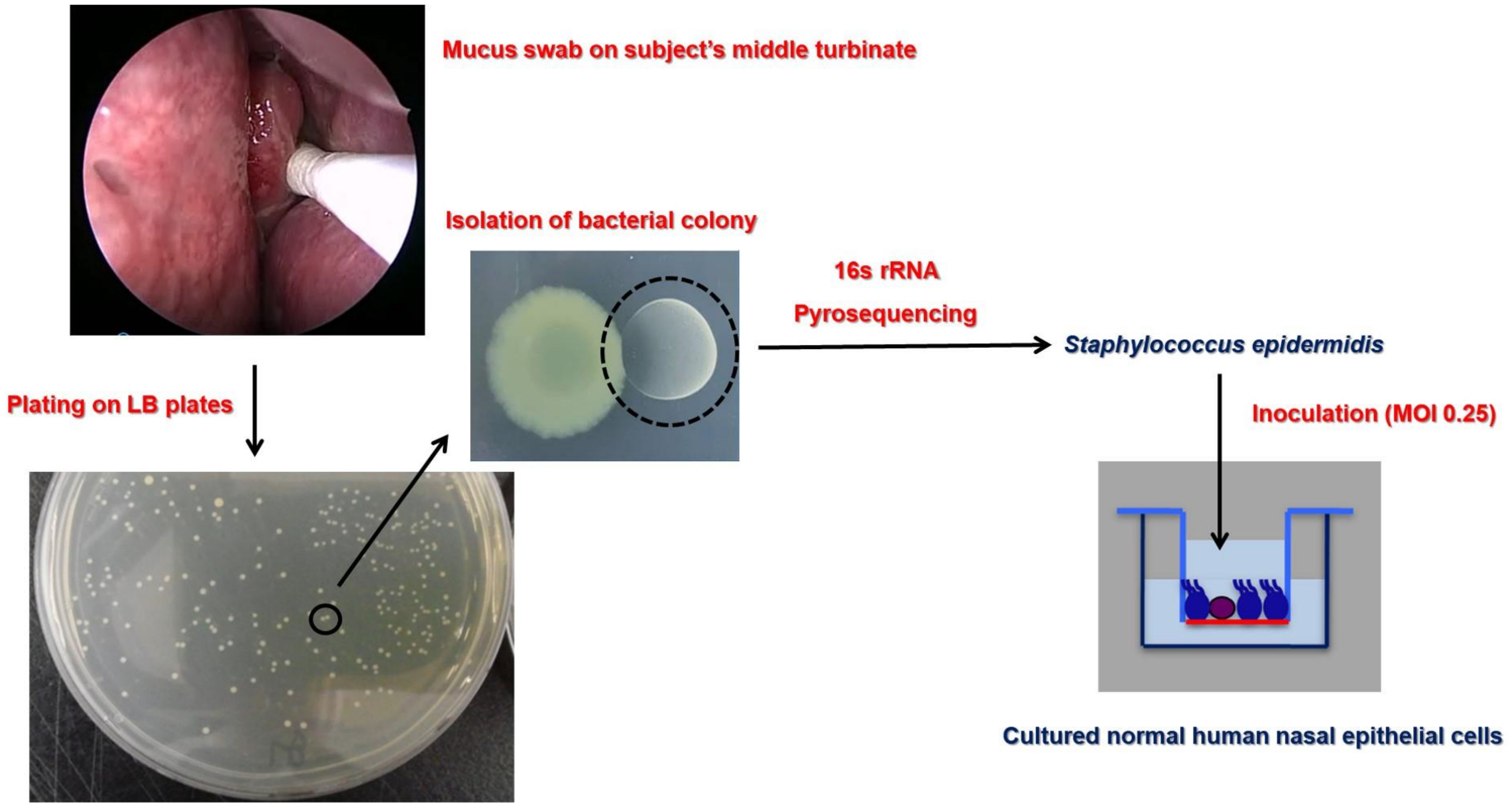

Supplementary figure 6 The full uncropped scans of the western blots included into the main documents

(A) Figure 2G

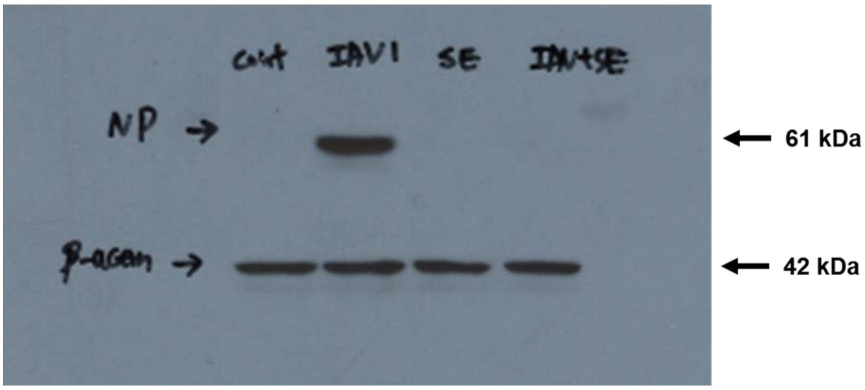

(B) Figure 2H

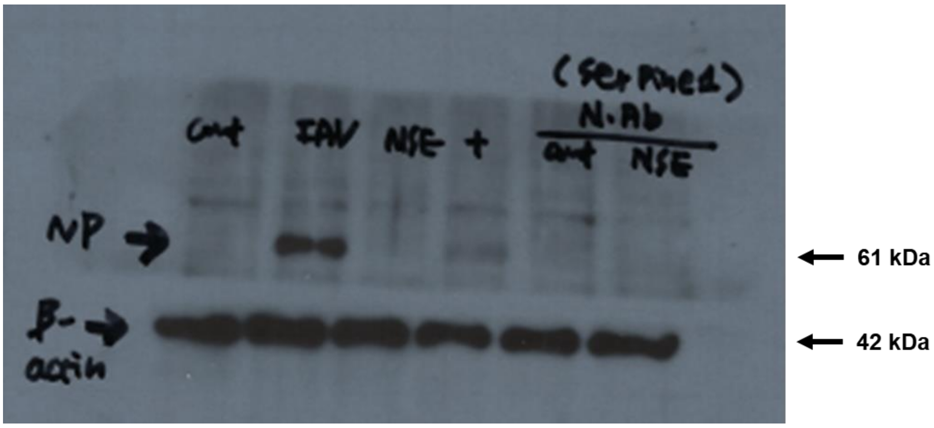

(C) Figure 3L

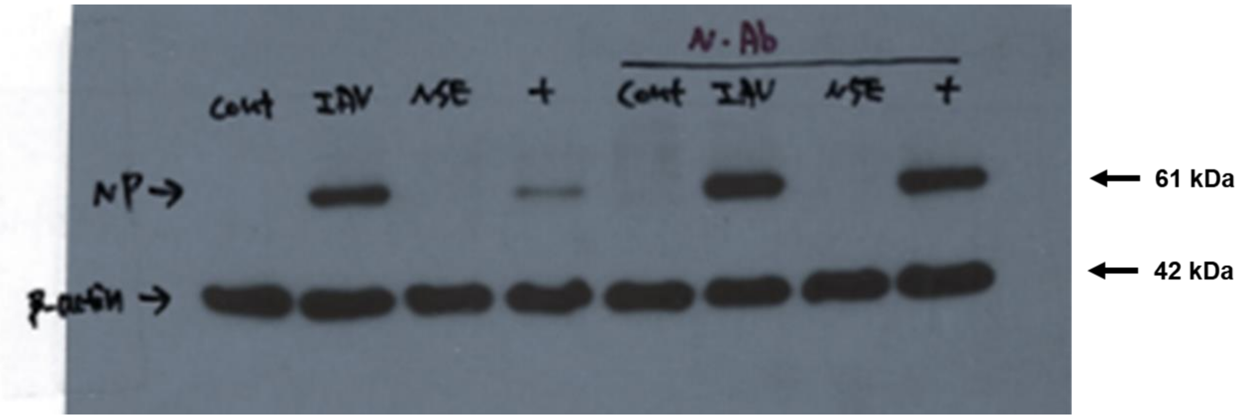

(D) Figure 4G

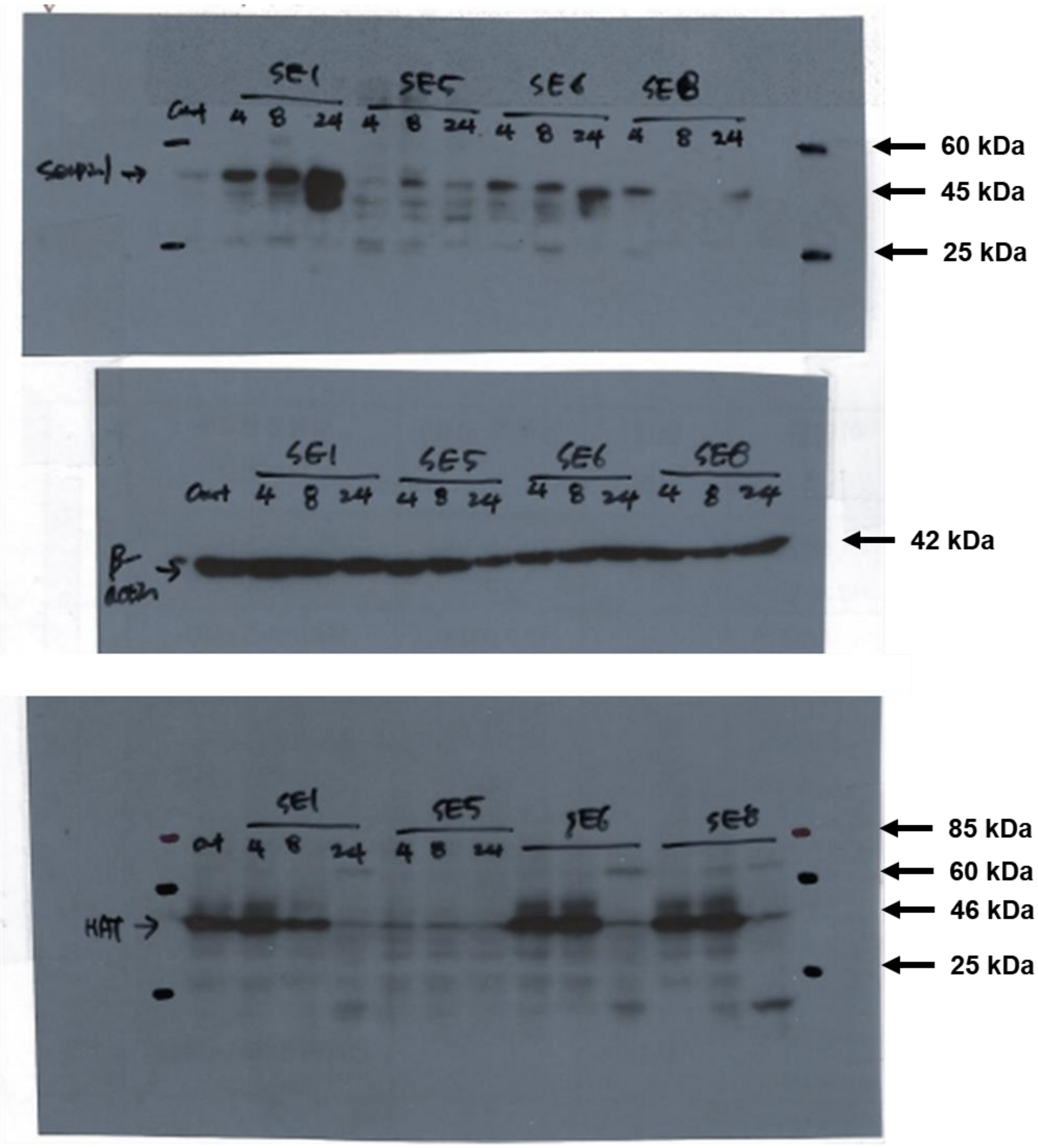

Supplementary table 1. The list of DEGs linked with biological processes enriched GO categories in *S. epidermidis*-inoculated NHNE cells (determined by DAVID analysis).

GO category	ID	Gene symbol	*S.epidermidis*/control (fold)
**Defense response to virus**	20989	**IL23A**	4.725
	19461	**DDIT4**	4.194
	771	**ZC3H12A**	3.217
	10611	**MICA**	3.12
	14656	**POLR3D**	3.12
	20074	**BNIP3**	3.041
	29546	**BPIFA1**	2.51
	33245	**IFNGR2**	2.439
	24881	**ISG20**	2.135
	12690	**TRIM56**	0.403
	27955	**TRIM25**	0.394
	6414	**DTX3L**	0.333
	13019	**ZC3HAV1**	0.314
	25456	**PLA2G10**	0.24
**Modulation by symbiont of entry into host**	32704	**LGALS1**	24.363
	7707	**CXCL8**	12.98
	12821	**CAV1**	5.171
	10694	**HLA-DRB1**	4.101
	18646	**TMPRSS4**	0.418
	27955	**TRIM25**	0.394
	27174	**LGALS9**	0.379
	10408	**TRIM38**	0.361
	19834	**TRIM8**	0.361

**Serine peptidase inhibitor**	5214	**SERPINE2**	40.804
	12691	**SERPINE1**	17.155
	5869	**COL7A1**	5.426
	9772	**SPINK1**	5.147
	9567	**SPOCK1**	5.062
	11097	**CD109**	3.635
	23566	**SERPINA3**	2.633
	29022	**SERPINA7**	2.482
	29016	**SERPINB5**	2.367
**Serine peptidase**	4659	**FAP**	11.843
	19503	**PLAU**	5.627
	18646	**TMPRSS4**	0.418
**Virus entry to host**	12821	**CAV1**	5.171
	20909	**ITGA5**	5.141
	30459	**ICAM1**	4.253
	22451	**EFNB2**	3.907
	24191	**VPS18**	3.219
	7043	**CLDN1**	3.008
	12326	**EGFR**	2.592
	22537	**GAS6**	2.193
	22184	**KPNA3**	0.492
	4654	**DPP4**	0.231

Supplementary table 2. Top 10 significant GO Biological Process and DEGs in *S. epidermidis*-inoculated NHNE cells (determined by ErichR analysis).

term	p-value	overlap_genes
**positive regulation of cytokine production** (GO:0001819)	1.27E-12	GBP5.CD274.IL33.CCBE1.CSF2.PRKDC.GATA3.HLAG. TRIM6.RGCC.NIRP10.IL23A.LEP.IRF1.PDE4B.CHI3L1. FFAR2.IL6ST.TLR5.TLR3
**cellular response to cytokine stimulus** (GO:0071345)	2.41E-12	GBP5.CEF2.PNPT1.CCL22.CCL20.IL1R2.DOCK8. RC3H1.GATA3.CBL.MMP9.IL1F10.TRIM6.SOCS1. IL23A.IRF1.IL2RB.ANKRD1.CHI3L1.VIM.SOX9.IL6ST.GBP1
**cytokine-mediated signaling pathway** (GO:0019221)	1.01E-11	CSF2.PELI2.IFIT5.GATA3.CBL.SAMHD1.IL1RL1.SOCS1. GBP1.IL33.CCL22.CCL20. IL1R2.HLA-GMMP9.IL17RB.IL1F10.IL23A.LEP.IRF1. IL2RB.VIM.IL6ST.MAP3K14.IL17C
**regulation of interleukin-8 production** (GO:0032677)	1.96E-07	NLRP10.C5AR2.LEP.OTUD7B.CHI3L1.FFAR2.TLR5.TLR3
**regulation of inflammatory response** (GO:0050727)	5.52E-07	IL33.NLRP10.IK23A.NAPEPLD.S100A12.FFAR2.GATA3.MMP9. VAMP2.TLR3.BRD4
**neutrophil degranulation** (GO:0043312)	9.09E-07	RAB3A.RAB5C.DDX3X.SERPINA1.CALML5.ATG1.KRT1.IQGAP2. MMP9.OSCAR.GLIPR1.DOK3.LRG1.S100A12.CHI3L1.VAMP2
**positive regulation of intracellular signal transduction** (GO:1902533)	1.01E-06	DDX3X.PELI2.IFIT5.GATA3.FGF1.CBL.TRIM6.IL23A.LEP. REL.S100A12.CHI3L1.SOX9. MAP3K14.IKBKE.TLR3.BRD4
**neutrophil activation involved in immune response** (GO:0002283)	1.01E-06	RAB3A.RAB5C.DDX3X.SERPINA1.CALML5.ARG1.KRT1. IQGAP2.MMP9.OSCAR. GLIPR1. DOK3.LRG1.S100A12. CHI3L1.VAMP2
**neutrophil mediated immunity** (GO:0002446)	1.10E-06	RAB3A.RAB5C.DDX3X.SERPINA1.CALML5.ARG1.KRT1. IQGAP2.MMP9.OSCAR.GLIPR1. DOK3.LRG1.S100A12.CHI3L1.VAMP2
**regulation of T cell proliferation** (GO:0042129)	1.88E-06	CD274.RIPK3.ARG1.IL23A.LEP.IL6ST.HLA-G

Supplementary table 3. The list of DEGs linked with GO category: Serine type endopeptidase inhibitor in *S. epidermidis*-inoculated secretory and suprabasal NHNE cells (determined by DAVID analysis).

GO: Serine type endopeptidase inhibitor	ID	Gene symbol	*S.epidermidis*/control (fold)
**Secretory cells**	2938	**AGT**	0.4
	5869	**COL7A1**	2.851
	9567	**SPOCK1**	2.042
	9772	**SPINK1**	4.227
	12691	**SERPINE1**	14.413
	29022	**SERPINB7**	2.163
	29722	**WFDC5**	3.796
	29724	**PI3**	2.052
	29740	**WFDC6**	0.44
**Suprabasal cells**	5214	**SERPINE2**	2.122
	5869	**COL7A1**	3.374
	11097	**CD109**	2.592
	12562	**TFPI2**	2.702
	12691	**SERPINE1**	12.387
	29016	**SERPINB5**	2.273
	29022	**SERPINB7**	3.107
	29722	**WFDC5**	4.721
	29737	**WFDC2**	0.377
